# HIF1A-repressed PUS10 regulates NUDC/Cofilin1 dependent renal cell carcinoma migration by promoting the maturation of miR-194-5p

**DOI:** 10.1186/s13578-023-01094-4

**Published:** 2023-08-18

**Authors:** Wenqin Luo, Zhehao Xu, Huan Wang, Zeyi Lu, Lifeng Ding, Ruyue Wang, Haiyun Xie, Qiming Zheng, Yudong Lin, Zhenwei Zhou, Yang Li, Xianjiong Chen, Gonghui Li, Liqun Xia

**Affiliations:** Department of Urology, Sir Run Run Shaw Hospital, Zhejiang University School of Medicine, Hangzhou, 310016 China

**Keywords:** PUS10, Renal cell carcinoma, microRNA, NUDC, HIF-1A

## Abstract

**Background:**

Renal cell carcinoma (RCC) is characterized by a high rate of distant metastasis, which leads to poor prognosis in patients with advanced RCC. PUS10 has been recognized as a member of the pseudouridine synthase family, and recently other functions beyond the synthesis of the RNA modification have been uncovered. However, little is known about its role in diseases such as cancer.

**Methods:**

RT-qPCR, western blot and immunohistochemistry were used to measure the expression of PUS10 in RCC tissues. Transwell assay, wound healing assay, and in vivo metastasis model were conducted to determine the function of PUS10 in RCC progression. MicroRNA sequencing and GEO database were used to screen for the downstream microRNAs of PUS10. RNA immunoprecipitation, dual luciferase reporter assay, immunostaining, and rescue experiments were employed to establish the PUS10/miR-194-5p/nuclear distribution protein C(NUDC)/Cofilin1 axis in RCC migration. Chromatin immunoprecipitation and dual luciferase reporter assay were used to verify its upstream transcriptional regulator.

**Results:**

The expression of PUS10 was significantly decreased in RCC tissues, and low expression predicted poor prognosis. In vitro and in vivo experiments showed that PUS10 suppressed RCC migration, which, however, was independent of its classical pseudouridine catalytic function. Mechanically, PUS10 promoted the maturation of miR-194-5p, which sequentially inhibited RCC migration via disrupting NUDC-dependent cytoskeleton. Furthermore, hypoxia and HIF-1 A were found involved in the downregulation of PUS10.

**Conclusion:**

We unraveled PUS10 restrained RCC migration via the PUS10/miR-194-5p/NUDC/Cofilin1 pathway, which independent of its classical catalytic function. Furthermore, a linkage between the critical tumor microenvironment hallmark with malfunction of the forementioned metastasis inhibition mechanism was presented, as demonstrated by repressed expression of PUS10 due to hypoxia and HIF-1A.

**Supplementary Information:**

The online version contains supplementary material available at 10.1186/s13578-023-01094-4.

## Background

As one of the most common malignancies in the urinary system, renal cell carcinoma (RCC) brings a great social burden and accounts for approximately 4% of adult cancer cases [1]. Nephrectomy is recognized as an effective treatment for RCC patients with localized tumors. However, its high rate of metastasis and invisibility at its early stages result in 20–30% of patients presenting with distant metastasis at their initial diagnosis [2], which, combined with its resistance to chemotherapy and radiation therapy [3], leads to a poor prognosis. The last decade has witnessed the evolution of treatment for advanced RCC, as the application of receptor tyrosine kinase (RTK) inhibitor drugs such as sunitinib and pazopanib greatly improves the disease-free survival of advanced RCC patients. However, their efficacy decreases as tumors develop drug resistance after 6–15 months of therapy in most patients [4]. Thus, there is an urgent need to investigate the underlying mechanism of RCC metastasis and identify new targets to expand the treatment options of advanced RCC.

Pseudouridine (Ψ), one of the most ubiquitous RNA modifications called ‘the fifth nucleotide’, was first discovered in the 1950s [5]. As a modification deposited in various RNAs, it can impact multiple aspects of RNA biology, including splicing, degradation and translation [6–8]. Its ‘writer’ is composed of two categories, dyskerin (DKC1) and the pseudouridine synthase (PUS) family. While DKC1 synthesizes pseudouridine with snoRNA, PUSs, including PUS1-10 in eukaryotes, independently catalyze the reaction [9, 10]. As epigenetics has become a focus in oncology, scientists have begun to explore the role of pseudouridine and its synthases in tumor progression. Cui et al. reported that PUS7 promotes the proliferation and self-renewal of glioblastoma multiforme cells by depositing pseudouridylation on tRNAs and sequentially regulating protein translation [11]. DKC1 was revealed to accelerate colon cancer cell proliferation by stabilizing the mRNA of target ribosomal proteins by adding pseudouridine [12]. However, as a newly identified member in the PUS family producing Ψ54 in tRNAs [13], PUS10 has rarely been studied in oncology. On the other hand, some investigations disclosed the functions of PUS enzymes beyond their catalysis of pseudouridine [14, 15]. PUS10 has been demonstrated to be an indispensable modulator of TRAIL-induced apoptosis [16], and very recently, Yi et al. established its novel function in promoting the maturation of miRNA in collaboration with microprocessors, namely, DGCR8 and DROSHA [17].

MicroRNAs (miRNAs) are a group of small noncoding RNAs consisting of 19 to 25 nucleotides that posttranscriptionally regulate the expression of genes in the form of RNA-induced silencing complexes (RISC) [18–20]. In the past few years, the popularization of miRNA profiling has boosted the exploration of miRNAs. In the field of oncology, emerging evidence suggests that miRNAs play a significant role in cancer progression and possess great potential as diagnostic biomarkers and therapeutic targets [21, 22]. In renal cell carcinoma (RCC), abundant miRNAs were reported to be differentially expressed in tumors and could influence proliferation, metastasis and drug resistance. For instance, Hill et al. reported that miR-200 family members could target ZEB1 and ZEB2 to regulate epithelial-mesenchymal and mesenchymal-epithelial transition [23]. miR-194-3p was uncovered to facilitate cell proliferation, migration and sunitinib resistance by suppressing ARID1A [24]. Some miRNAs were validated in patient cohorts and could serve as biomarkers to predict prognosis [25, 26]. However, compared to the expanding understanding of these miRNAs and their mRNA targets, limited knowledge has been obtained about how the expression of miRNAs is dysregulated in cancer.

The tumor microenvironment (TME) has drawn great research attention and has been identified to facilitate cancer progression in diverse aspects [27]. Apart from the sophisticated crosstalk between cancer cells and adjacent nonneoplastic cells, one of the most significant factors in the TME of most solid tumors is hypoxia, caused by the rapid proliferation of cancer cells [28]. Intratumoral hypoxia also triggers hypoxia inducible factor (HIF) signaling and sequentially influences tumor development in angiogenesis, reprogramming metabolism, epithelial-mesenchymal transition (EMT), immune evasion and so on [28, 29]. In renal cell carcinoma, HIF signaling is even more essential due to the frequent inactivation mutation of von Hippel‒Lindau (VHL) in sporadic cases. The absence of protein VHL (pVHL) boosts the accumulation of HIFs and their transcriptional impact [30]. While the aforementioned mechanisms have been revealed, new clues are still required to present the full picture of the role of HIFs in cancer progression.

In this study, we identified a novel member in the pseudouridine synthase family, PUS10, that is downregulated in RCC tumors at the mRNA and protein levels based on public databases and the SRRSH RCC cohort. We illustrated that it acts as a tumor suppressor in RCC by inhibiting the migration of cancer cells both in vivo and in vitro, which, however, is independent of its RNA modification function. Previous research has reported its nonclassical functions in promoting miRNA processing. According to our microRNA sequencing and online data, we revealed that PUS10 could facilitate the maturation of miR-194-5p and subsequently regulate NUDC/Cofilin1-dependent cytoskeleton dynamics to inhibit RCC cancer migration. Finally, we demonstrated that hypoxic conditions and HIF-1 A activation might lead to the decreased expression of PUS10 in RCC tissues.

## Methods

### Tissue samples

Renal cell carcinoma specimens and its adjacent normal tissues are collected by the department of Urology, Sir run run shaw hospital, Zhejiang university school of medicine between 2017 and 2022. Their application to our research is approved by Ethics Committee of SRRSH. The clinical characteristics of all patients are presented in Additional file 1: Table S1. Informed consent is obtained from all patients.

### Cell cultures

Four RCC cell lines used in our research, Caki-1, 786-O, OS-RC-2 and ACHN were purchased from Cell Bank of Type Culture Collection of the Chinese Academy of Sciences. 786-O, OS-RC-2 were cultured in RPMI-1640 with 10% FBS (Cellmax, China), and the Caki-1 cell line was cultured in McCoy 5 A medium with 10% FBS (Cellmax), ACHN was cultured in MEM medium containing 10% FBS (cellmax). Cells were cultured at 37 °C with 5% CO_2_. For hypoxia incubation, a 1% O_2_ environment was created in hypoxia chamber.

### microRNA sequencing

Two pairs of RCC and paratumor normal tissues were obtained by Department of Urology, Sir Run Run Shaw Hospital. Sample preparation was conducted based on the instruction of Illumina NextSeq500’s. The total RNA was ligated to 3’ and 5’ small RNA adapters. After amplification, the PCR products were selected by size using PAGE gel according to instructions of NEBNext® Multiplex Small RNA Library Prep Set for Illumina® (Illumina, USA). The constructed library was qualified and then sequenced by HiSeq 2500 (Illumina). Differentially expressed microRNAs were determined by edgeR package, with a cut off threshold of log2 fold change > 2 and FDR < 0.01.

### Cell transfection

The cells were seeded in 6 well plates. For transient transfection, when the confluence reached 30–40%, siRNAs or microRNA mimics/inhibitors was transfected into RCC cells using the RFect siRNA/miRNA Transfection Reagent (Baidai, China) according to the manufacturer’s instructions. siRNAs were synthesized by Genepharma (China) with all sequences exhibited in Additional file 2: Table S2. For ectopic expressing, plasmids containing wild type PUS10 and mutant PUS10 were transfected into cells when the confluence reached 50% using a Lipofectamine 3000 kit (Invitrogen, Thermo Fisher Scientific, United States) according to the manufacturer’s instructions. These plasmids are designed and produced by GeneChem (China). The PUS10 knockdown lentivirus were designed, synthesized and collected by GeneChem (China) to construct stable PUS10 knockdown cell lines using transfection reagent provided by GeneChem (China), puromycin (Selleck, China) were utilized to maintain knockdown efficiency.

### RNA extraction and qRT-PCR

Cells and tissues were lysed with their total RNA extracted using Trizol reagent (CWBio, China). The concentration of total RNA was measured with Nanodrop 2000 (Thermo Fisher, United States). A total of 500ng RNA was used for cDNA synthesis using HiFiScript RT (CWBio). The miRNA 1st strand cDNA synthesis kit (MR101, Vazyme) was utilized to amplify the identified microRNA with specific stem-loop primers. Normal cDNA was produced using the HiFiScript cDNA Synthesis Kit (CWBio). mRNA and microRNA expression levels were quantified by conducting qRT-PCR on a Roche Light Cycler 480 instrument with the SYBR Green (CWBio). The primer sequences are listed in Additional file 3: Table S3.

### Western blot and antibodies

Cells and tissues were lysed using RIPA extraction reagent (Beyotime, China), the proteins were denatured at 100 °C for 30 min. After that, proteins (10 µg) were added into the wells of 12% SDS–polyacrylamide gel and separated, then transferred onto PVDF membranes (Bio-Rad, Hercules, USA) and blocked in 5% nonfat milk. The membranes were incubated with primary antibodies overnight at 4 °C and secondary antibodies for 2 h, an Femto-ECL chemiluminescence kit (Fdbio, Hangzhou, China) was sequentially used to visualize the protein bands. All information of our antibodies applicated in this research is listed in Additional file 3: Table S3.

### Immunohistochemistry (IHC)

IHC was performed in patient-derived specimens. Tissues were fixed in 4% paraformaldehyde and embedded in paraffin then dewaxed and rehydrated. After blocking, the slides were incubated with PUS10 antibody (#NBP2-48941, Novus) overnight at 4 °C then a secondary antibody for 1 h at room temperature. The sections were stained by DAB for following histological analysis.

### Bioinformatics

GEPIA (http://gepia.cancer-pku.cn/) was used to determine the prognostic value of PUS10 in KIRC and the correlation between the expression of PUS10 with that of microprocessor proteins. The GEO dataset GSE53757 was downloaded from Gene Expression Omnibus at NCBI (https://www.ncbi.nlm.nih.gov/geo/). Human Protein Atlas (https://www.proteinatlas.org/) provided us with PUS10 IHC figures in kidney cancer tissues. LinkedOmics (http://www.linkedomics.org/login.php) database was used to obtain RCC prognosis related microRNAs. cBioPortal (https://www.cbioportal.org/) was utilized to identify the correlations of PUS10 and other downstream genes based on TCGA data. And Oncomine (https://www.oncomine.com/) database was utilized to determine the correlation between the microRNAs and PUS10; Targetscan (http://www.targetscan.org/vert_72/) and miRDB (http://mirdb.org/) were refered to predict the target of microRNA; Heatmap were generated with OmicStudio tools (https://www.omicstudio.cn/tool), and Venn diagram were draw by webtools (http://bioinformatics.psb.ugent.be/webtools/Venn/).

### Transwell assay

The ability of cell migration was evaluated by conducting transwell assay using 8-µm pore filters (Millipore, Germany). RCC cells suspended in serum-free RPMI1640 or McCoy5a were seeded into the upper chamber with the lower chamber filled with 15% FBS medium. After 24 h, the cells pass the pores and attached on the membrane’s lower side were fixed by 4% paraformaldehyde, stained with crystal violet then counted in three randomly chosen fields under microscope.

### Wound healing assay

Caki-1 and 786-O cells were seeded into 6-wells plates. When the confluence reached 100%, we scratched the cell monolayer carefully using a 200 µL pipette and photographed it as 0 h. Subsequently, cells were cultured in serum-free medium for 24 h and photographed to examine their migration ability based on wound healing rate.

### Animal model

5-week-old Balb/c nude mice were divided into two groups and a total of 5 × 10^5^ luciferase-labeled ACHN cells suspended in 50 µL PBS with or without PUS10 knockdown were respectively injected into their tail vein. After 8 weeks, mice were anesthetized and their metastasis was imaged using in vivo imaging system (IVIS).

### Dot blot

A total of 250 ng RNA was denatured at 95 °C for 3 min and dropped onto a N+ membranes (GE Health, USA). The RNA was cross-linked to the membrane under UV light. Subsequently, the membrane was washed using PBS containing 0.02% Tween to remove the uncrosslinked RNA, then blocked with 5% nonfat milk, and incubated with an pseudouridine antibody overnight at 4 °C. After that the membrane was incubated with a secondary antibody and the dots were visualized with ECL chemiluminescent detection system. 0.02% methylene blue was used to reflect the total amount of RNA.

### RNA immunoprecipitation (RIP)

The RIP assay was conducted to identify the enrichment of primary microRNA on PUS10 or DGCR8 with Magna RIP Kit (Millipore, USA) according to the manufactures’ instruction. Briefly, 2 × 10^7^ cells were collected and lysed in RIP lysis buffer. After centrifugation at 4 °C, the supernatant was collected to incubate with specific antibodies and magnetic beads at room temperature. The beads-antibody complex was then washed several times, before he RNA was finally eluted and extracted. The amount of indicated RNA was then quantified by RT-qPCR.

### Chromatin immunoprecipitation (ChIP)

ChIP experiments were performed with ChIP assay kit (Cell signaling Technology, USA) according to the manufacturer’s guidelines. Briefly, 5 × 10^6^ RCC cells were cross-linked with 1% formaldehyde. Subsequently, the nuclear precipitate was fragmented through enzymatic digestion and sonication. After centrifugation, the supernatant was collected and incubated with identified antibodies overnight at 4 °C. Then, the chromatin antibody complex was de-crosslinked and the enriched DNA was purified and quantified by qRT–PCR.

### Luciferase report assay

A luciferase reporter vector containing wild-type or mutant 3ʹUTR of NUDC were constructed. 293 T cells were seeded in a 24-well plate and transfected with luciferase vector and miR-194-5p mimics or NC. After 48 h, cells were lysed and the luciferase activity was measured using the Dual-Luciferase Reporter Assay System (Promega). Renilla luciferase activity was measured as a reference.

Another luciferase reporter vector incorporating wild-type or mutant promoter region of PUS10 were constructed. luciferase vector and siRNA targeting HIF1A/HIF2A or NC were co transfected to 293T cells, following steps are same as above.

### Immunostaining

Cells seeded on coverslips were fixed in 4% paraformaldehyde for 10 min, permeabilized using 0.1% Triton X-100, then incubated with Cofilin1 antibody (10960-1-AP, proteintech, China) overnight at 4 °C. The cells were then incubated with secondary antibody for 2 h at room temperature. After that, cells were stained by FITC-conjugated/rhodamine-conjugated phalloidin (Thermo Fisher, USA). The nuclear was stained with DAPI (Thermo Fisher, USA). The signals were detected with a fluorescence microscope (Leica, Wetzlar, Germany).

### Fluorescence in situ hybridization (FISH)

Cy3-labeled pri-miR-194 probe was designed and synthesized by GenePharma (Shanghai, China). The experiment was performed with a FISH kit (Genepharma) according to the manufactures’ guidelines. The signals were visualized using the fluorescence microscope (Leica, Wetzlar, Germany).

### Statistics

Results in our study are presented as the mean ± SD and were analyzed using GraphPad prism7 (GraphPad Software, Inc., CA). The statistical difference between two groups was measured by a two-tailed Student’s t test. Statistical significance was defined as *P value < 0.05, **P value < 0.01, ***P value < 0.001.

## Results

### PUS10 is downregulated in renal cell carcinoma and associated with clinical prognosis

**Fig. 1:**
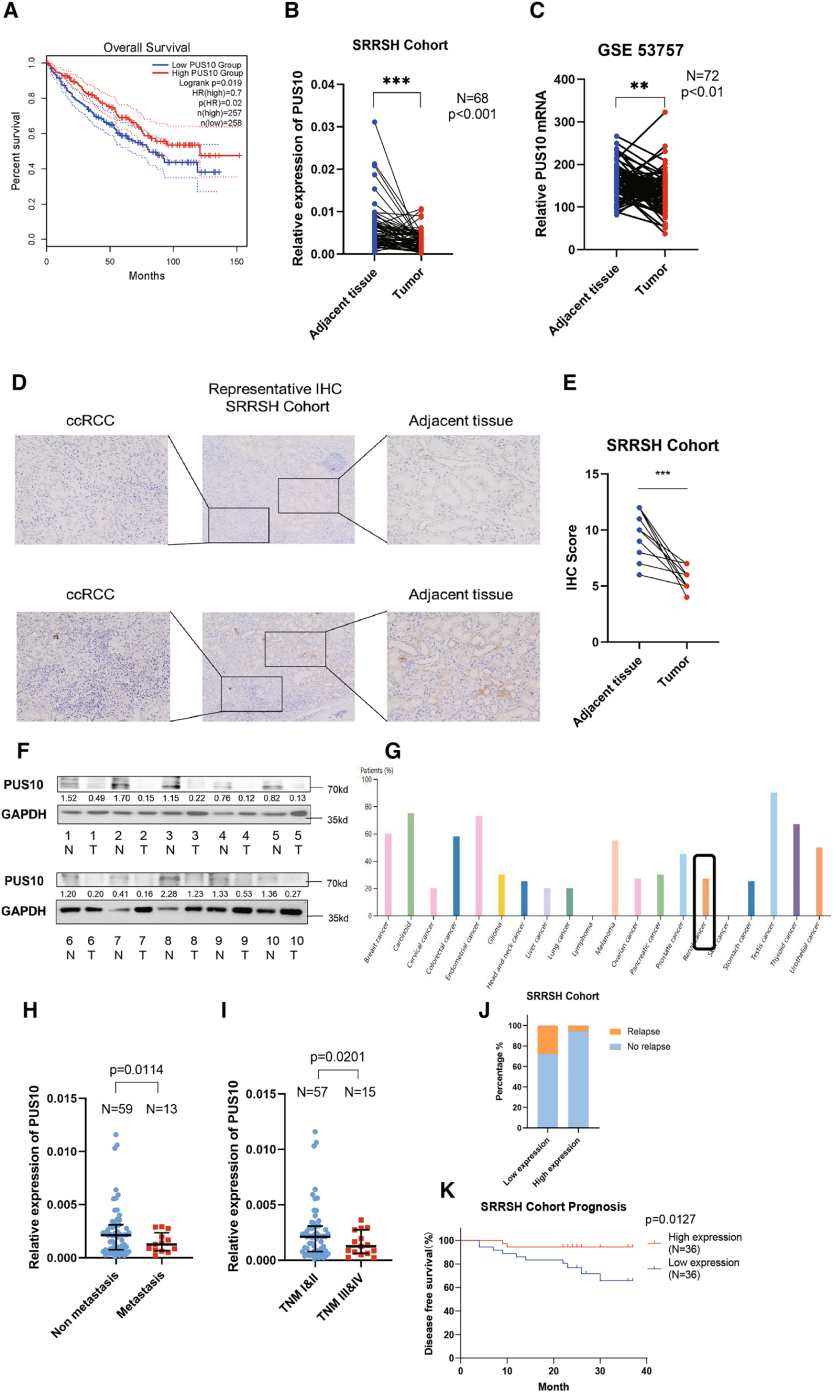
PUS10 is decreased in RCC, and low expression of PUS10 predicts poor prognosis. **A** Kaplan‒Meier analysis showed the correlation between the expression of PUS10 and the overall survival of patients based on the TCGA cohort. **B** RT‒qPCR revealed the relative expression of PUS10 in 68 paired RCC tumors and normal tissues from our SRRSH cohort, normalized to GAPDH. **C** Expression of PUS10 in paired RCC tumor and normal tissue sequencing data in GSE53757. **D** Representative IHC images of PUS10 in 10 pairs of RCC tissues in the SRRSH cohort. **E** The IHC scores of each section were calculated. Dots, IHC score; lines, pairs of normal and tumor tissues. SRRSH, Sir Run Run Shaw Hospital. **F** Western blot assay showing the protein level of PUS10 in 10 pairs of RCC tissue from our cohort. **G** Pancancer analysis of the PUS10 level in multiple cancers based on the Human Protein Atlas database. **H** Relative expression of PUS10 in RCC tissues with or without metastasis. **I** Relative expression of PUS10 in RCC tissues with different TNM stages. **J** The frequency of relapse in RCC patients with low and high expression of PUS10, the low and high PUS10 expression groups were cut off by the median expression.** K** Kaplan‒Meier analysis showed the difference of disease free survival between low and high PUS10 expression groups. *P < 0.05, **P < 0.01, ***P < 0.001; ns, not significant. Data are representative of three independent experiments

Pseudouridine synthases are a group of enzymes responsible for the synthesis of the RNA modification pseudouridine (Ψ). While their functions have been gradually elucidated [7, 31], research focusing on their effect on tumorigenesis is limited. Herein, we investigated the role of PUS10 in renal cell carcinoma. Kaplan‒Meier survival analysis of TCGA data using GEPIA (http://gepia.cancer-pku.cn/) indicated that low expression of PUS10 predicted a poorer prognosis in RCC patients (Fig. 1A), suggesting that it might act as a tumor suppressor. By performing qRT‒PCR using our SRRSH RCC cohort specimen, we identified the downregulation of PUS10 in tumor tissue compared with adjacent normal tissue (Fig. 1B). Consistently, *in silico* exploration with GEO published sequencing data GSE53757 using 72 pairs of tumor and matched normal tissues validated its repressed expression in KIRC (Fig. 1C) [32]. Its downregulation in RCC was further confirmed by IHC and western blot results using our RCC samples (Fig. 1D–F). While RCC cancer cells originate from kidney tubular endothelial cells, our IHC results exhibited a dramatic contrast in PUS10 staining between these two types of cells, hinting that PUS10 possibly plays a significant role in the evolution of RCC. Similar results were observed in IHC figures in the Human Protein Atlas (Additional file 4: Figure S1A). In addition, a pancancer analysis implied the relatively low expression of PUS10 in kidney cancer compared with other cancers (Fig. 1G). With these findings, it is safe to conclude that the expression of PUS10 was inhibited in RCC.

Given the significant downregulation of PUS10 expression, we were prompted to explore the correlation between its expression level and the clinical parameters of RCC. By investigating clinical information, we identified a lower expression of PUS10 in patients with tumor metastasis (Fig. 1H). In addition, compared with low grade tumors, tumors considered in TNM stage III and IV exhibited decreased expression of PUS10 (Fig. 1I). Furthermore, a higher frequency of tumor relapse in patients from PUS10 low expression group was revealed (Fig. 1J) and Kaplan–Meier plotter analysis of the SRRSH RCC cohort disclosed that patients with decreased PUS10 expression had shorter disease free survival (Fig. 1K). These results confirmed the potential value of PUS10 as a novel biomarker in RCC prognosis prediction.

### Knockdown of PUS10 significantly promotes RCC cancer migration

**Fig. 2:**
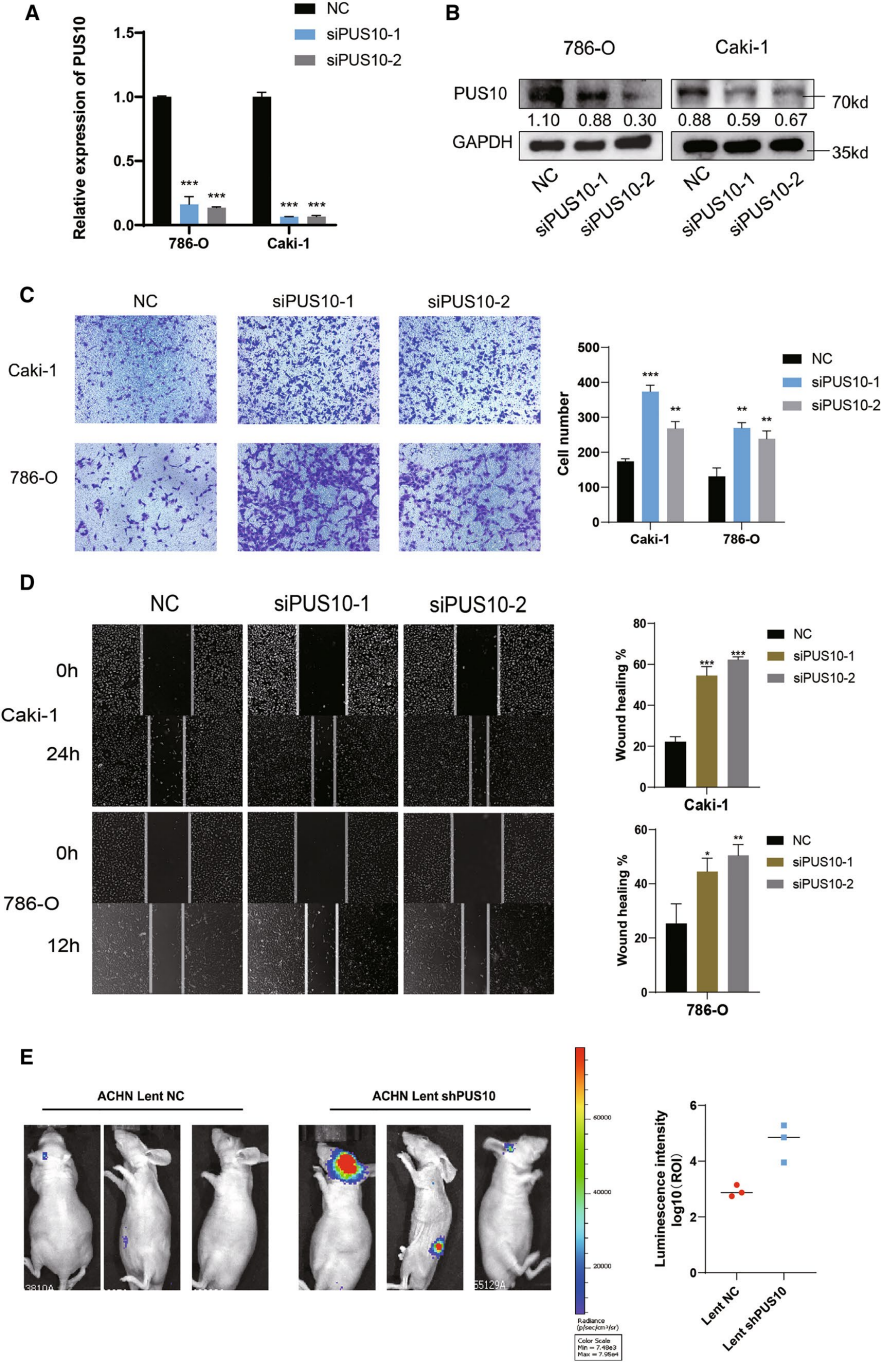
Silencing of PUS10 promotes RCC migration in vitro and in vivo. **A** qRT‒PCR assay revealed that PUS10 was knocked down in 786-O and Caki-1 cells after the transfection of siRNAs. Three independent experiments are shown as the mean ± SD, and GAPDH was used as a reference. **B** Representative western blot images showing the silencing efficiency of PUS10 in RCC cell lines. **C** Transwell assays and wound healing assays were performed to measure the migration ability in vitro after PUS10 depletion. Migrating cells in three replicate experiments were counted and are presented as the mean ± SD in the histogram. **D** Wound healing assay validated the promoted migratory ability of 786-O and Caki-1 cells after PUS10 knockdown. A representative of three replicated experiments is shown. The wound healing rate was calculated and presented as the mean ± S.D. **E** Luciferase-tagged ACHN cells with or without PUS10 knockdown were constructed and injected into the tail vein of nude mice. Bioluminescent images were taken after 8 weeks with signal intensities (photons/s/cm^2^/sr) qualified using the IVIS system. *P < 0.05, **P < 0.01, ***P < 0.001; *ns* not significant

To investigate whether PUS10 could act as a tumor suppressor in RCC, we silenced PUS10 in 786-O and Caki-1 cells using two siRNAs, and the knockdown efficiency was examined at the mRNA and protein levels (Fig. 2A and B, Additional file 4: Figure S2A). CCK-8 assays were adopted to measure the ability to proliferate, and no significant difference was detected (Additional file 4: Figure S2D). A previous study revealed that PUS10 mediates TRAIL-induced cell apoptosis [16, 33], but without external inducement, little distinction in apoptosis was observed (Additional file 4: Figure S2E). However, in transwell and wound healing assays, PUS10 knockdown led to significantly enhanced RCC cell migration (Fig. 2C and D, Additional file 4: Figure S2B, C). To verify its effect in vivo, we established luciferase-expressing ACHN cells with stable PUS10 knockdown and constructed a metastasis model by injecting knockdown and corresponding control cells into the tail vein of nude mice. PUS10 silencing was shown to promote renal cell carcinoma metastasis with a dramatically stronger bioluminescent signal (Fig. 2E).

### PUS10 inhibits RCC cell migration in a pseudouridine-independent manner

**Fig. 3:**
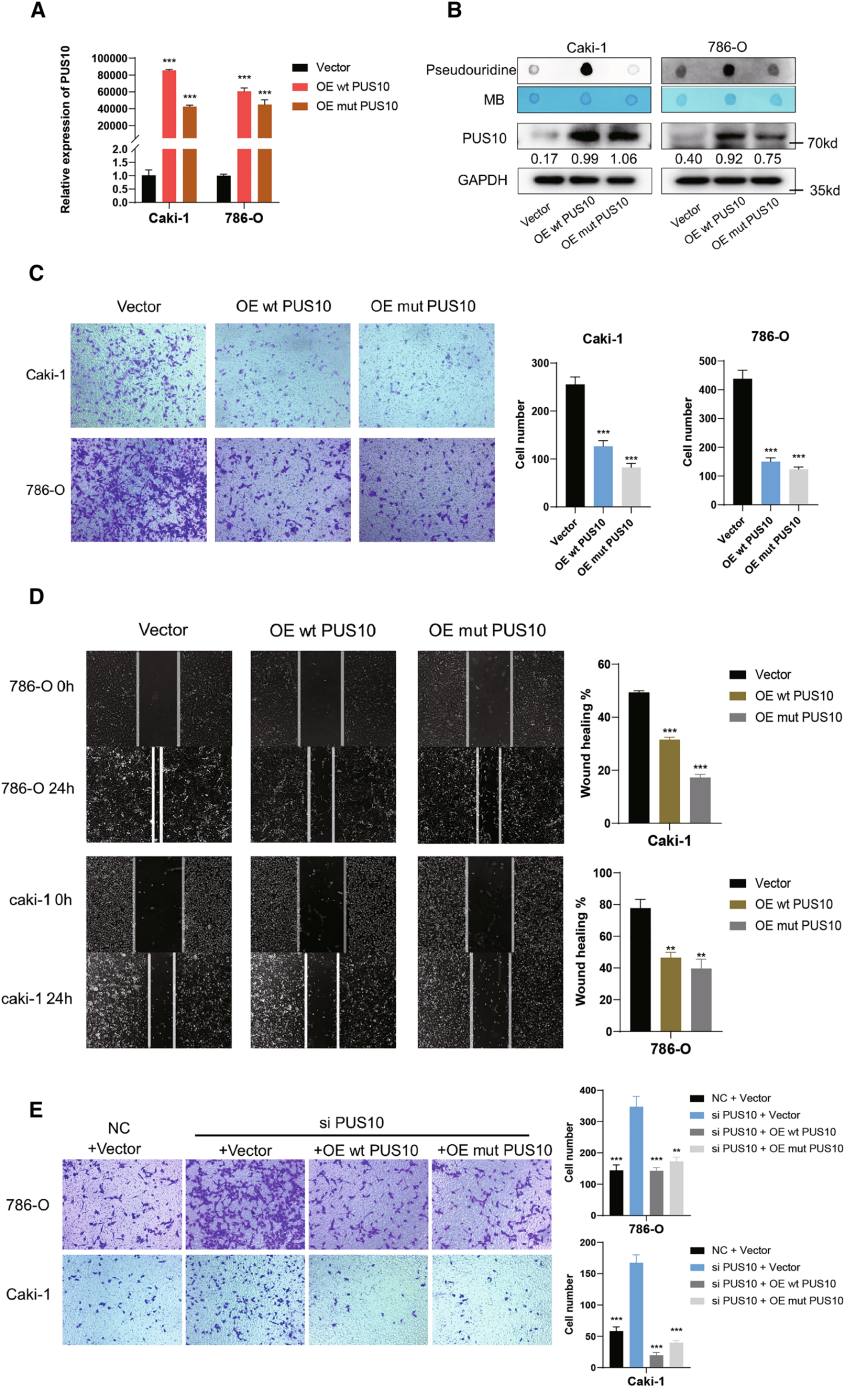
PUS10 inhibits the migration of RCC in a catalytic-independent manner. **A** Overexpression of PUS10 was verified by qRT‒PCR using GAPDH as a reference. **B** Western blot and dot blot assay were performed to verify the construction of wildtype and mutant PUS10 overexpressing models in vitro*.* **C** Transwell assays were conducted to evaluate the migration inhibition effect of wild-type and catalytically incompetent PUS10. Migrating cells in three replicate experiments were counted and are presented as the mean ± SD in the histogram. **D** Wound healing assay validates the similar impact of wild-type and mutant PUS10 ectopic expression on the migratory ability of RCC cells. **E** Transwell assays showed that supplementation with either wild-type or mutant PUS10 could reverse the promotion of RCC migration induced by PUS10 silencing with siRNA. Migrating cells in three replicate experiments were counted and are presented as the mean ± SD in the histogram. *P < 0.05, **P < 0.01, ***P < 0.001; *ns* not significant

To further validate the role of PUS10 in RCC metastasis, we generated a PUS10-overexpressing cell model (Fig. 3A and B, Additional file 4: Figure S3A). After transfection with the PUS10 overexpression plasmid, RCC cell lines displayed significantly inhibited migration. (Figure 3C and D, Additional file 4: Figure S3B, C). To explore whether the function of PUS10 is mediated by the fluctuation of pseudouridine modification on RNA, we constructed a catalytically incompetent PUS10 overexpression plasmid with a D344A mutation. Dot blots were performed to confirm that the mutant plasmid would not alter the pseudouridine level as the wild-type plasmid. (Fig. 3B). Intriguingly, a similar impact on RCC cell migration was observed when mutant PUS10 was ectopically expressed (Fig. 3C and D, Additional file 4: Figure S3B, C). Moreover, compensation with either wild-type or catalytically disabled PUS10 efficiently rescued the enhanced migration induced by PUS10 silencing (Fig. 3E). Previous research has demonstrated the synthase activity of PUS10 is restricted in cytoplasm [13]. However, in RCC cancer cells, by immunofluorescent assays, we found that it was mainly distributed in the nucleus (Additional file 4: Figure S3D), which also suggested that altered tRNA modification might not be the primary mechanism explaining the phenotype. Taken together, we inferred that PUS10 inhibits RCC cell migration independent of its pseudouridine catalytic function.

### PUS10 enhances RCC migration by promoting mir-194-5p maturation

**Fig. 4:**
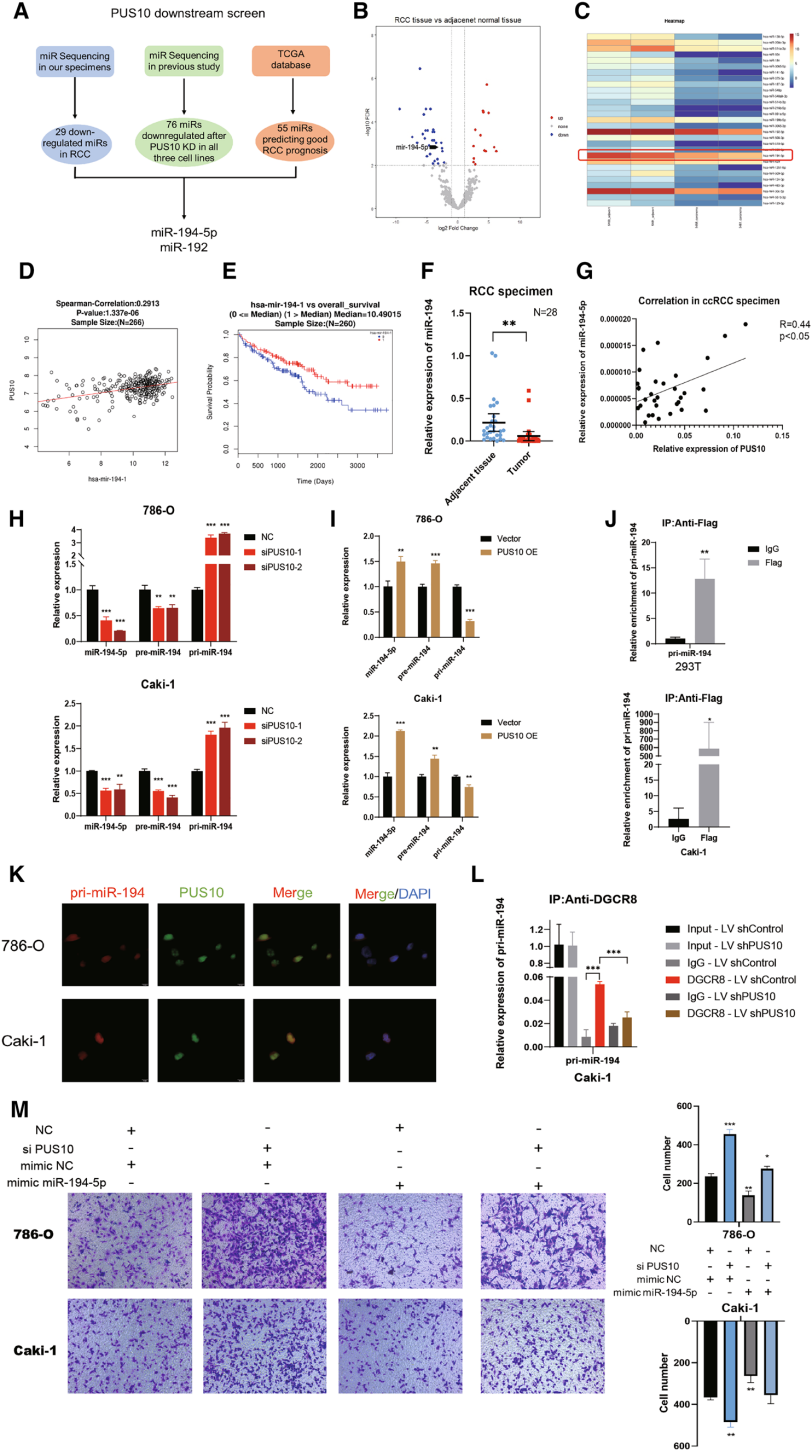
PUS10 inhibits RCC migration by promoting the maturation of miR-194-5p. **A** Schematic of our downstream candidate screening strategy. **B** Volcano plot of our microRNA sequencing results showing differentially expressed microRNAs in RCC tissues compared with adjacent normal tissues. (|log_2_FC| > 1 and *FDR* < 0.01) Twenty-nine downregulated (blue) microRNAs in RCC were identified. **C** Heatmap showing the downregulation of 29 microRNAs in our tissue. **D** Pearson correlation analysis demonstrated that the expression of miR-194-5p was positively correlated with that of PUS10 in 266 RCC tissues according to TCGA database. (*r* = 0.2913, *P* value < 0.0001). **E** Kaplan‒Meier analysis showed the correlation between the expression of miR-194-5p and the overall survival of patients based on the TCGA cohort. **F** The decreased expression of miR-194-5p in RCC was verified in SRRSH patient-derived tissues by performing qRT‒PCR. **G** A positive correlation between the expression of miR-194-5p and PUS10 was identified in the SRRSH cohort. (*r* = 0.44, *P* value < 0.05). **H** qRT‒PCR assay showed that the expression of miR-194-5p and pre-miR-194 were repressed after the transfection of siRNA targeting PUS10 in 786-O and Caki-1 cells, while their primary precursor pri-miR-194 accumulated. **I** qRT‒PCR assay showed that the expression of miR-194-5p and pre-miR-194 were increased after the ectopic expression of PUS10 in RCC cells, with their precursor pri-miR-194 being further consumed. **J** The interaction between PUS10 and pri-miR-194 was determined by performing an RNA immunoprecipitation (RIP) assay and qRT‒PCR with an anti-Flag antibody in Caki-1 and 293T cells transfected with a flag-tagged PUS10 plasmid. **K** Immunofluorescence (IF) and fluorescence in situ hybridisation (FISH) assays exhibited the co-localization of PUS10 and pri-miR-194 in the nucleus of RCC cells. **L** RIP assays showed that the enrichment of pri-miR-194 on the microprocessor protein DGCR8 was impaired by the depletion of PUS10 in Caki-1 cells. **M** Compensation of miR-194-5p in Caki-1 and 786-O cells abolished the enhanced migration induced by PUS10 knockdown. Migrating cells in three replicate experiments were counted and are presented as the mean ± SD in the histogram. *P < 0.05, **P < 0.01, ***P < 0.001; *ns* not significant

Browsing published literature, a study delivered by Yi, etc. got our attention, in which they revealed a novel noncatalytic function of PUS10, interacting with a microprocessor to regulate the maturation of a portion of microRNAs [17]. Considering that mounting research has demonstrated the dysregulation of microRNAs and the crucial role they play in RCC, we speculated that PUS10 might influence cancer migration in a manner dependent on its downstream microRNAs. The strong correlation between the mRNA level of PUS10 and that of established microRNA biogenesis-associated proteins, such as DICER, DGCR8, AGO1 and AGO3, in KIRC tumor tissues hinted at the involvement of PUS10 in microRNA processing (Additional file 4: Figure S4A). Looking into the microRNA sequencing data upon PUS10 silencing in a previous study[17], we found that several downregulated microRNAs are related to RCC prognosis. Further combined with our microRNA sequencing data using 2 pairs of RCC specimens, miR-194-5p and miR-192, two prognosis-related microRNAs repressed in RCC, stand out as downstream candidates that could be regulated by PUS10 (Fig. 4A C). Our screening strategy was confirmed by the expression of both microRNAs being positively correlated with that of PUS10 in KIRC tissues (Fig. 4D, Additional file 4: Figure S4B). Both microRNAs have been established by existing research as metastasis biomarkers [34] and possess prediction value in RCC (Fig. 4E, Additional file 4: Figure S4C). Our sequential verification of their function revealed that miR-194-5p inhibited cancer cell migration, as shown by Transwell and wound healing assays (Additional file 4: Figure S4D–F). Thus, miR-194-5p was selected as the potential functional target of PUS10. Using our SRRSH RCC specimens, we identified the downregulation of miR-194-5p and its coexpression with PUS10 in tumor tissues by performing qRT‒PCR (Fig. 4F and G).

To validate that PUS10 promotes the expression of miR-194-5p, we transfected PUS10-targeting siRNAs into 786-O and Caki-1 cells. Sequentially, we detected the decreased expression of miR-194-5p and pre-miR-194, while its precursor pri-miR-194 was upregulated (Fig. 4H). The ectopic expression of PUS10 resulted in the opposite trend (Fig. 4I). These findings suggested that PUS10 regulated the transition from pri-miR-194 to pre-miR-194 and mature miR-194-5p. Subsequently, we explored whether PUS10 regulates the expression of miR-194-5p by promoting the splicing of its precursor driven by microprocessor [35]. Thus, we performed an RNA immunoprecipitation (RIP) assay with an anti-Flag antibody in cells transfected with flag-tagged PUS10 plasmid and demonstrated the binding between PUS10 and pri-miR-194 (Fig. 4J). RNA fluorescence in situ hybridisation (FISH) and immunofluorescence assay supported their interaction by exhibiting the co-localization of PUS10 and pri-miR-194 in the nucleus (Fig. 4K). Furthermore, we conducted another RIP assay using an anti-DGCR8 antibody. In line with our assumption, when PUS10 was silenced, the enrichment of pri-miR-194 on DGCR8, a recognized microprocessor protein, was reduced (Fig. 4L). These results implied that PUS10 participates in the processing of micro194 by facilitating the interaction between pri-miR-194 and the microprocessor. In addition, rescue experiments showed that supplementation with miR-194-5p obviously reversed the enhanced migration induced by PUS10 knockdown (Fig. 4M). Taken together, in this part, we inferred that PUS10 exerts its anti-tumour effect by promoting the maturation of miR-194-5p.

### NUDC served as the downstream target of miR-194-5p

**Fig. 5:**
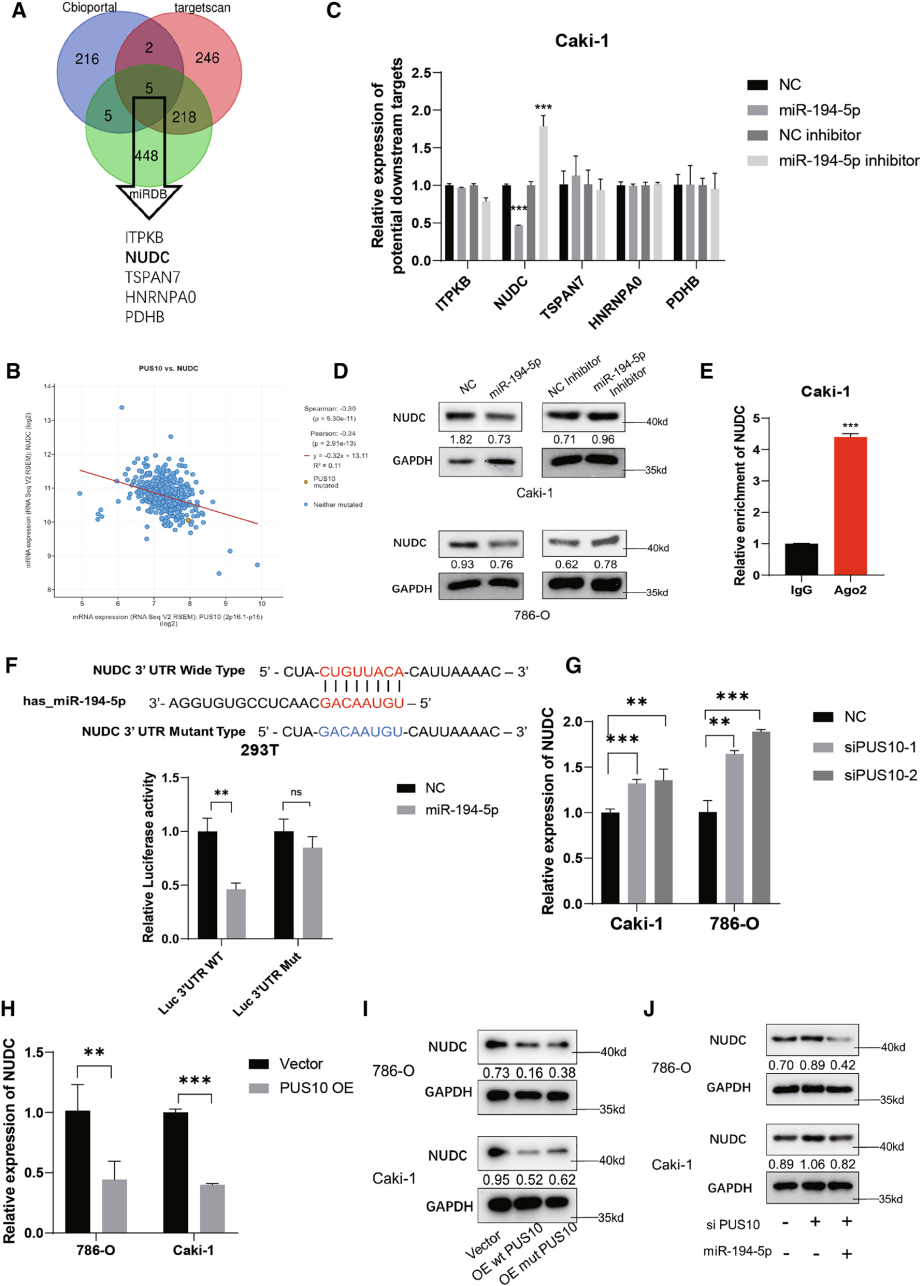
miR-194-5p targets NUDC in RCC. **A** Schematic of our strategy to identify the downstream target of miR-194-5p. **B** Pearson correlation analysis demonstrated that the expression of NUDC was negatively correlated with that of PUS10 in KIRC based on the TCGA database. (*r* = − 0.34, *P* value < 0.0001). **C**, **D** Changes in the expression of NUDC at the mRNA and protein levels upon transfection of miR-194-5p mimics and inhibitor in RCC cells were determined by qRT‒PCR and western blotting. Data from three independent experiments are presented as the mean ± SD in the histogram. **E** RIP assays were conducted to demonstrate the enrichment of NUDC on an anti-Ago2 antibody, suggesting that its degradation might depend on Ago2 and microRNAs. Data from three independent experiments are presented as the mean ± SD in the histogram. **F** Dual luciferase reporter assays were conducted by transfecting plasmids containing the WT or Mut 3ʹUTR of NUDC and miR-194-5p or control mimics into HEK-293T cells. The relative luciferase activity was normalized to Renilla luciferase activity. Data from three independent experiments are presented as the mean ± S.D. **G**, **H** qRT‒PCR was performed to measure the changed expression of NUDC mRNA upon the silencing or ectopic expression of PUS10 in Caki-1 and 786-O cells. Data from three replicated experiments are presented as the mean ± SD in the histogram. **I** Western blot results reflected the repressed expression of NUDC upon the ectopic expression of wild-type or mutant incompetent PUS10 in Caki-1 and 786-O cells. **G** Western blot assay showed that the increased expression of NUDC induced by PUS10 depletion could be reversed by miR-194-5p mimics. *P < 0.05, **P < 0.01, ***P < 0.001; *ns* not significant

MicroRNAs were established to bind with the 3ʹUTR of their mRNA targets to influence their stability. To elucidate the detailed mechanism by which miR-194-5p functions to inhibit migration, we used TargetScan and miRDB, two online microRNA target prediction databases. By overlapping the predicted targets with genes that possess a negative correlation with PUS10 in KIRC tumors, we screened five possible downstream genes, ITPKB, NUDC, TSPAN7, HNRNPA0 and PDHB (Fig. 5A, B). Among them, NUDC was finally chosen because its expression was decreased after ectopic expression of miR-194-5p and increased when the microRNA was inhibited (Fig. 5C, Additional file 4: Figure S5A). Consistently, a similar trend was observed in the western blot results (Fig. 5D). In addition, an anti-Ago2 RIP assay in Caki-1 cells indicated the enrichment of NUDC mRNA on Ago2, suggesting that it undergoes microRNA- and Ago2-mediated degradation (Fig. 5E). To confirm that miR-194-5p regulates the expression of NUDC by binding to its 3’UTR, a dual luciferase reporter assay using luciferase plasmids containing the wild-type or mutant NUDC 3’UTR sequence was conducted. Transfection of miR-194-5p decreased the luciferase activity in the wild-type group but caused little change in the mutant group (Fig. 5F). In accordance with our expectation, at the mRNA level, the expression of NUDC was also regulated by PUS10 (Fig. 5G, H). At the protein level, our western blot results revealed that it could be similarly suppressed by either wild-type PUS10 or mutant PUS10 (Fig. 5I). Moreover, the increased NUDC expression induced by PUS depletion was rescued by miR-194-5p ectopic expression (Fig. 5J). Thus, we determined that NUDC is downstream of miR-194-5p and regulated by PUS10 and miR-194-5p in RCC.

### NUDC/Cofilin1 mediated the repressed migration induced by PUS10

**Fig. 6:**
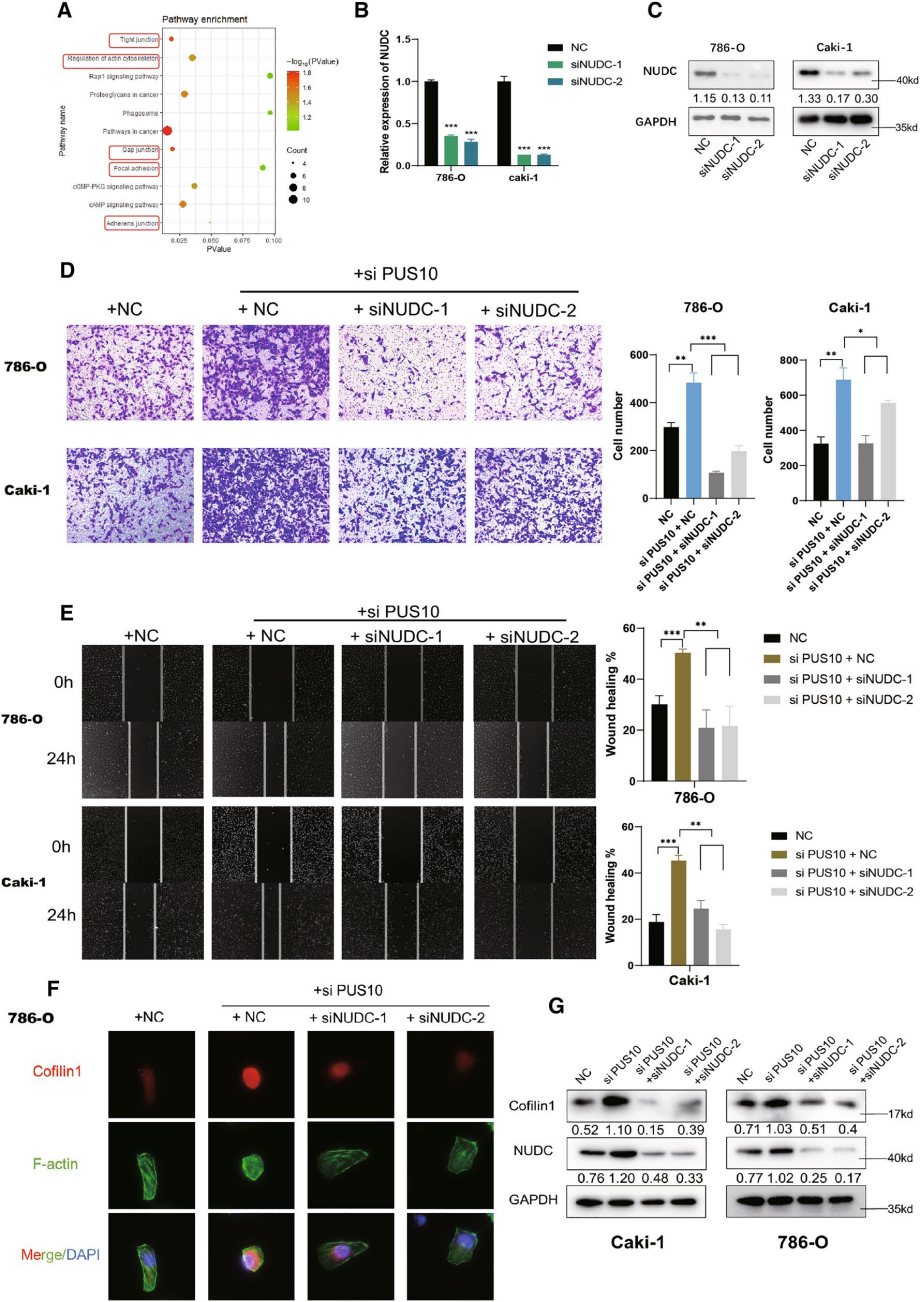
The NUDC/cofilin1-dependent cytoskeleton is involved in the PUS10-induced migration suppression of RCC. **A** KEGG enrichment analysis of the genes possessing a negative correlation with PUS10 in KIRC. **B**, **C** The efficiency of NUDC silencing was demonstrated at the mRNA and protein levels. **D** Transwell assays revealed that NUDC depletion abrogated the promoted migration caused by PUS10 knockdown in Caki-1 and 786-O cells. Migrating cells in three replicate experiments were counted and are presented as the mean ± SD in the histogram. **E** Wound healing assays showed that NUDC depletion reversed the enhanced wound healing rate induced by PUS10 knockdown in Caki-1 and 786-O cells. Three replicate experiments were conducted, and the results are presented as the mean ± SD in the histogram. **F** Representative images of immunostaining using anti-cofilin1 antibody and phalloidin are shown, showing the impact of PUS10 on the cofilin1-dependent cytoskeleton. **G** Western blot assays showed that the increased expression of Coflin1 induced by PUS10 knockdown was rescued by NUDC depletion in Caki-1 and 786-O cells. *P < 0.05, **P < 0.01, ***P < 0.001; *ns* not significant

By conducting KEGG analysis of the genes negatively correlated with PUS10 in KIRC tumors, several pathways associated with cell movement, such as tight junctions, regulation of actin cytoskeleton, and gap junctions, were enriched (Fig. 6A). NUDC has been illustrated to influence cell motility by interacting with Cofilin1, a key regulator of the cytoskeleton that is in charge of severing F actin [36]. To investigate whether NUDC mediates PUS10-induced migration inhibition, we performed transwell and wound healing assays. As the NUDC knockdown efficiency was validated (Fig. 6B, C), we found that depletion of NUDC could effectively rescue the enhanced cell migration after PUS10 knockdown (Fig. 6D, E). In cancer cells, F actin dynamics are indispensable to its locomotion, wherein ADF/Cofilin drives the disassembly of F actin [37]. Thus, we examined whether the PUS10/miR-194-5p/NUDC axis influences the cytoskeleton in RCC. Immunostaining and western blotting demonstrated higher expression of Cofilin1 in PUS10-silenced RCC cells, which could be reversed by NUDC knockdown (Fig. 6F, G, Additional file 4: Figure S6A). Consistently, actin stained by phalloidin tended to be depolymerized upon PUS10 knockdown and was prone to be reorganized to form stress fibers when NUDC was depleted (Fig. 6F, Additional file 4: Figure S6A). Taken together, we inferred that NUDC/cofilin1-dependent cytoskeleton dynamics mediate the augmented cancer migration activated by decreased PUS10.

### HIF-1A transcriptionally inhibited the expression of PUS10 in RCC

**Fig. 7:**
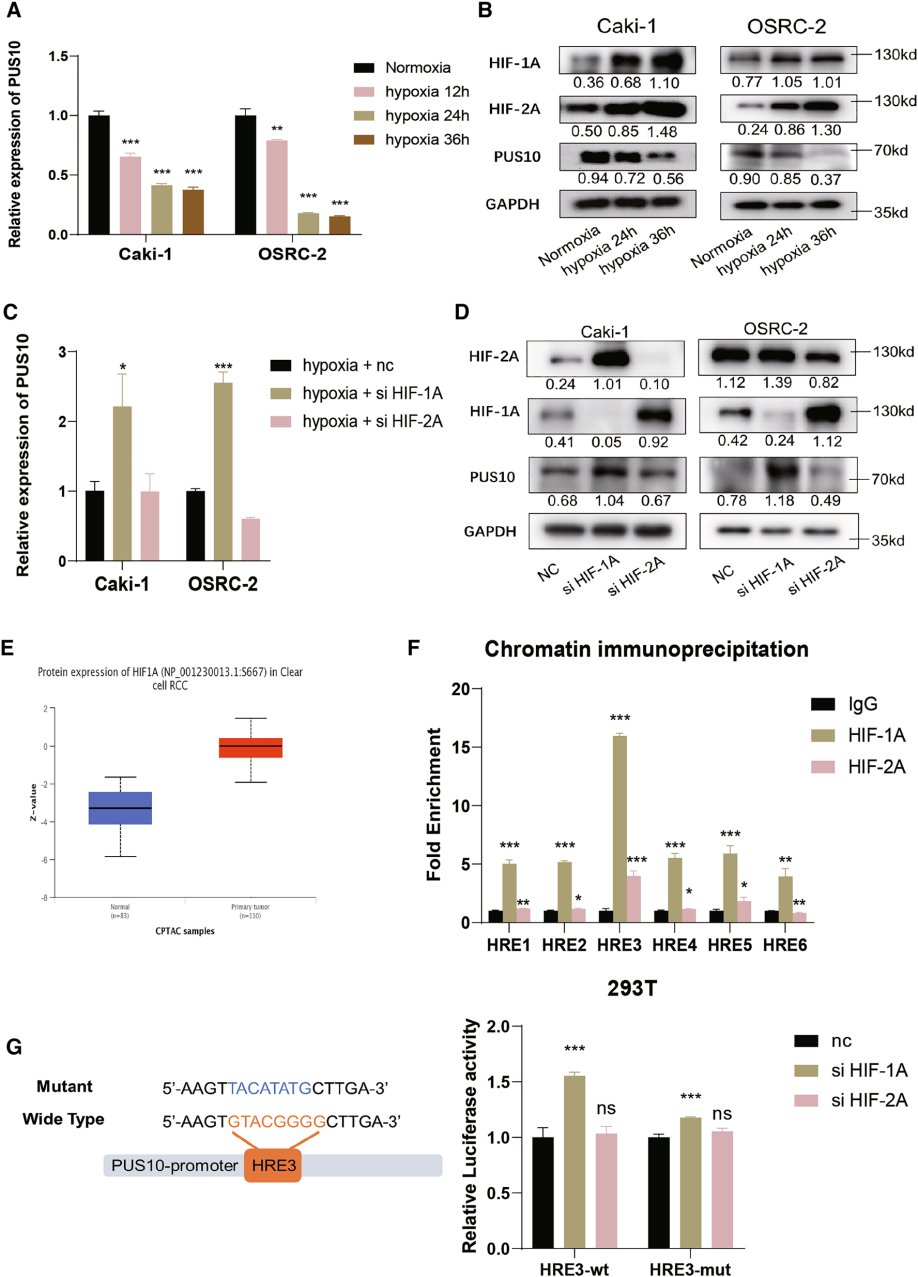
HIF-1A drives the downregulation of PUS10 in a transcriptional manner. **A**, **B** qRT‒PCR and western blotting showed that the expression of PUS10 was repressed at the mRNA and protein levels under hypoxia in Caki-1 and OS-RC-2 cell lines. **C**, **D** qRT‒PCR and western blotting showed that silencing HIF-1A but not HIF-2A increased the repression of PUS10 expression under hypoxia. **E** Online data from the Clinical Proteomic Tumor Analysis Consortium identified the accumulation of HIF-1A in KIRC. **F** Chromatin immunoprecipitation (ChIP) was performed to determine the binding between predicted binding sites on promoters and HIFs. **G** Dual luciferase reporter assays were performed by transfecting plasmids containing the WT or Mut promoter region of NUDC and siRNA targeting HIFs into HEK-293T cells. The relative luciferase activity was normalized to Renilla luciferase activity. Data from three independent experiments are presented as the mean ± S.D. *P < 0.05, **P < 0.01, ***P < 0.001; *ns* not significant

Furthermore, we questioned what led to the downregulation of PUS10 in RCC. Hypoxia and activated HIF signaling have been recognized as essential factors in cancer. In RCC, HIFs are involved in multiple aspects of tumor development and are promising drug targets [38–40]. To uncover whether hypoxia influences the expression of PUS10, the RCC cell lines Caki-1 and OS-RC-2 were exposed to 1% O2. PUS10 was inhibited under hypoxia at both the mRNA and protein levels, as HIFs accumulated (Fig. 7A, B). To examine whether PUS10 downregulation was attributed to HIFs, we silenced HIF-1A and HIF-2A in RCC cells under hypoxia. While HIF-2A knockdown resulted in little difference, HIF-1A depletion rescued the decreased expression (Fig. 7C, D), consistent with the accumulation of HIF-1A in KIRC tumor tissues (Fig. 7E). To further verify that HIF-1A repressed PUS10 expression as a transcription factor, we consulted the JASPAR database for the prediction of its binding sites on the PUS10 promoter and chose the top 6 for primer design. The enrichment of hypoxia response elements (HREs) on HIF-1A was demonstrated by chromatin immunoprecipitation (ChIP); among them, the enrichment of HRE3 was the highest (Fig. 7F). Thus, we constructed luciferase plasmids harboring the promoter of the PUS10 sequence with or without HRE3 mutation. The dual luciferase reporter assay showed that siHIF-1A but not siHIF-2A led to an elevation in luciferase activity in the control group, which was weakened in the mutant group (Fig. 7G), suggesting the key role played by HRE3 in the transcriptional regulation of HIF-1A. In this part, our results showed that the downregulation of PUS10 was at least partially ascribed to the accumulation of HIF-1A in RCC.

## Discussion

Pseudouridine synthase (PUS) enzymes are responsible for catalyzing the isomerization of uridine to pseudouridine on RNA [41]. In cancer research, PUS7 has received a lot of attention and has been reported to play a crucial role in the progression of leukemia, glioblastoma and colorectal cancer [11, 14, 42]. However, few published studies have focused on other PUS family members. PUS10 has been recognized as a pseudouridine synthase responsible for the Ψ54 and Ψ55 in tRNA [43], while recent researches disclosed its additional functions including mediating TRAIL-induced apoptosis and facilitating microRNA maturation independent of its pseudouridine synthase activity [16, 17]. Another latest study uncovered PUS10 was increased in aged hematopoietic stem and progenitor cells and diminished its reconstitution capacity independent of its pseudouridine catalytic function [44]. This versatility of PUS10 prompted us to explore its role in RCC progression.

In this study, we identified the decreased expression of PUS10 at the mRNA and protein levels in RCC and revealed the correlation between its expression and RCC prognosis. Functionally, silencing PUS10 significantly promoted the migration of RCC cancer cells in vitro and in vivo, certifying its tumor suppressor role in RCC. PUS10 has been demonstrated to be involved in TRAIL-induced apoptosis, whose nuclear export is accompanied by cytochrome c release and the activation of caspase-3 [16, 33]. But without exogenous TRAIL, we did not detect an obvious decline in RCC cell apoptosis after PUS10 knockdown. In addition, the deficient activation of caspase-9 in RCC might also account for the absence of the expected decreased apoptosis triggered by caspase-3 [45].

The direction of our mechanism exploration is determined by an intriguing phenomenon: As overexpressing PUS10 dramatically inhibits cancer cell migration, ectopic expression of the mutant PUS10, whose RNA modification function is abrogated, could have a similar inhibitory effect. This outcome indicates that the pseudouridine produced by PUS10 might contribute little to its tumor-suppressing impact.

Significant clues were obtained from the published work by Yi et al., in which PUS10 was shown to interact with the DROSHA-DGCR8 microprocessor and to be involved in the maturation of clusters of microRNAs independent of its catalytic activity [17]. Considering that mounting evidence has demonstrated the pivotal role of these small noncoding RNAs in regulating cancer development, it seems tempting to attribute the effect of PUS10 to the probable dysregulation of microRNAs in RCC.

To determine the potential downstream microRNA, we combined our microRNA sequencing data in RCC tissues with online sequencing results and sequentially performed in vitro experiments. After that, mir-194-5p stands out as a credible downstream target to mediate the effect of PUS10 in RCC. The connection between miR-194-5p and kidney cancer metastasis has been demonstrated by existing studies. A bioinformatic study revealed that miR-194-5p could be adopted to predict metastasis and disease-specific mortality [25]. Another study based on microRNA microarray analyses identified mir-194 as one of the metastasis-associated microRNAs that was downregulated in bone metastases of ccRCC patients compared with normal and primary tumor tissues [34]. In our work, we verified the functions of miR-194-5p in RCC cancer cell migration and investigated the cause of its downregulation in RCC. The processing and maturation of miRNAs have attracted great research attention in cancer research, and their abnormal fluctuation has been recognized to arouse the dysregulation of microRNAs. METTL3-induced N(6)-methyladenosine on primary microRNAs was significant in initiating DGCR8/DROSHA-mediated processing [46] and has been revealed to participate in diverse cancers [47, 48]. Another methyltransferase, METTL1, has also been shown to influence cancer progression based on its regulation of microRNA processing [49, 50]. Parallel with these studies, we identified another factor in this process, PUS10, that blocks cancer cell migration in RCC by facilitating the maturation of a functional microRNA.

Nuclear distribution protein C (NUDC) was first identified as a nuclear movement protein in filamentous fungi [51]. Subsequent studies discovered that it could interact with dynein/dynactin to form complexes and regulate various biological processes, such as mitosis and cell migration [52–54]. Zhou, etc., further reported its critical role in the actin cytoskeleton and ciliogenesis by binding to and stabilizing cofilin1 [36]. The dynamics of F-actin cytoskeleton assembly and disassembly are critical in cell locomotion [55]. Cofilin1, a small protein dispersed in the eukaryotic cytoplasm, can sever actin filaments and promote F-actin depolymerization to maintain the cytoskeleton dynamics of cells [37, 56]. In multiple studies in oncology, cofilin1 has been demonstrated to mediate tumor metastasis [57, 58]. In our work, by consulting an online database and conducting qRT-PCR and immunoblotting, we determined that the inhibitory effect of PUS10 and miR-194-5p on RCC is mediated by NUDC/Cofilin1-dependent F-actin dynamics, as reflected by phenotype rescue experiments and phalloidin staining results.

Hypoxia is one of the common traits in the tumor microenvironment and has been proven to be involved in tumor development. To cope with hypoxic stress, cancer cells adjust their inherent biological processes and employ multiple significant pathways, among which HIF signaling is recognized as the key player [28]. In RCC, the accumulation of HIFs is further boosted by the frequent loss of VHL [30]. Activation of HIF signaling has been demonstrated to have an essential influence on multiple aspects of renal cell carcinoma, including angiogenesis, metabolism and the cytoskeleton [59–61]. The small molecule HIF-2A antagonist belzutifan has been approved by the FDA for its therapeutic effect in clinical trials [62]. However, as a transcription factor can widely regulate downstream genes, its extensive impact is far from elucidated. Here, we determined that the downregulation of PUS10 is at least partially induced by HIF-1A by transcriptional inhibition, thus providing another link between hypoxia and RCC cancer metastasis.

## Conclusion

**Fig. 8:**
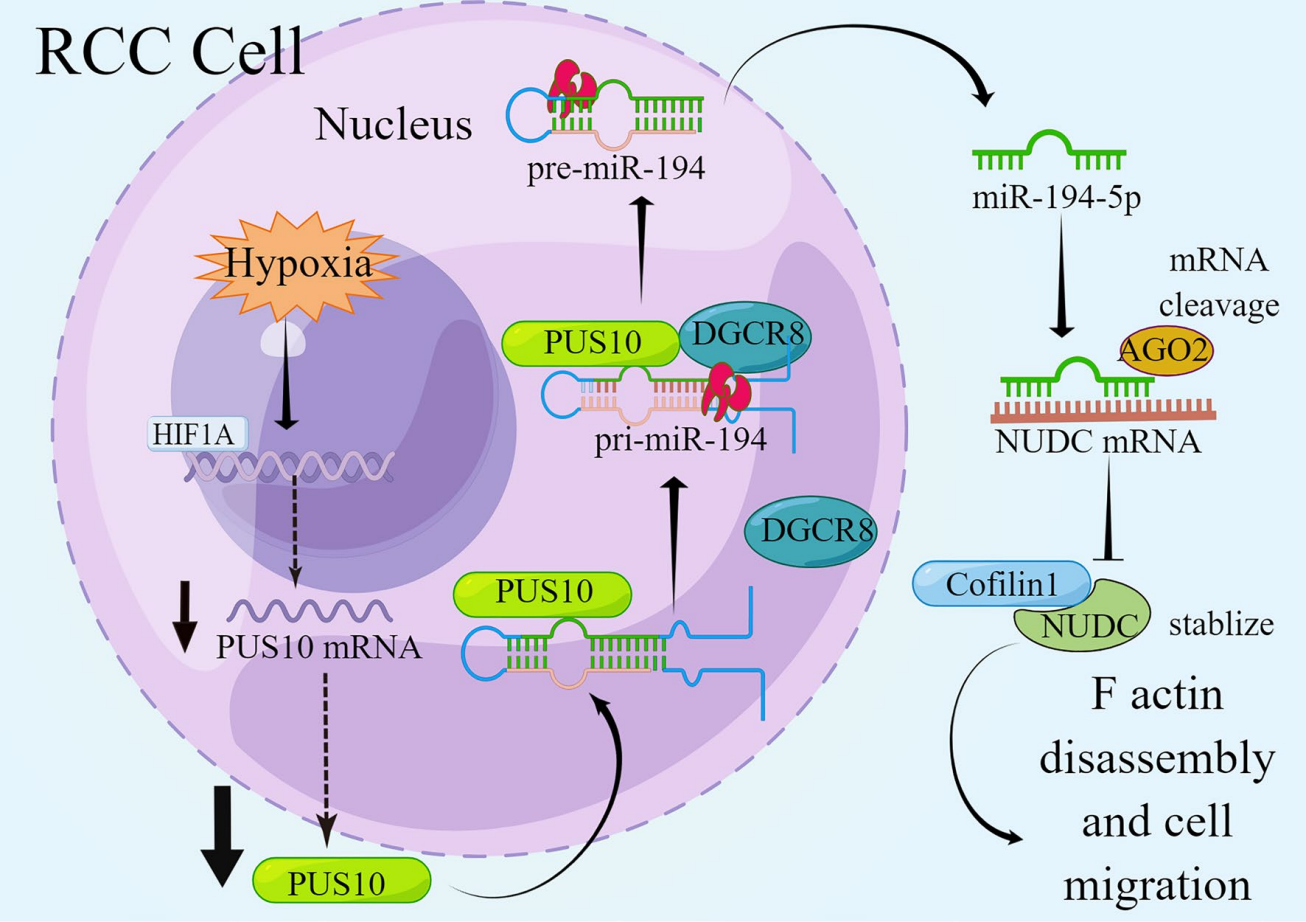
Schematic of our study illustrating the role of PUS10 in RCC (By Figdraw)

In summary, we demonstrated the decreased expression of PUS10 in RCC tumors and determined that it is a tumor suppressor that inhibits cancer cell migration. For the first time, we provide insight into PUS10 in cancer progression, which is not reliant on its RNA modification catalytic activity. To explore its mechanism, we established a PUS10/miR-194-5p/NUDC axis, ascribing the effect of PUS10 to the dysregulation of a key microRNA. Finally, we found that HIF-1A signaling might result in the silencing of PUS10 in cancer. Therefore, we presented a novel biomarker and potential treatment target in renal cell carcinoma to expand the therapeutic choices in advanced RCC cases (Fig. 8).

### Supplementary Information

**Additional file 1: Table S1.** Patients clinical informations.

**Additional file 2: Table S2.** Sequences of small interfering RNA used in this study.  

**Additional file 3: Table S3.** Primers and antibodies used in this study.

**Additional file 4: Figure S1.** Downregulation of PUS10 in RCC tissue at protein level in public IHC results. **Figure S2.** Knock down of PUS10 promotes cancer cell migration but doesn’t influence its proliferation and apoptosis. **Figure S3.** PUS10 inhibits RCC migration, which is not achieved by its pseudouridine synthase activity. **Figure S4.** miR-194-5p mediates the impact of PUS10 on RCC migration. **Figure S5.** NudC was identified as the downstream target of miR-194-5p. **Figure S6.** NudC/Cofilin1 was involved in PUS10 inducing inhibition on RCC migration.

## Data Availability

The datasets used during this research are available from the corresponding author on reasonable request.

## Abbreviations

RCC: Renal cell carcinoma
RT-qPCR: Reverse transcription-quantitative PCR
NUDC: Nuclear distribution protein C
DGCR8: DiGeorge Syndrome Critical Region 8
VHL: Von-Hippel-Lindau
HIF-1A: Hypoxia inducible factor-1A
HRE: Hypoxia response element
TCGA: The cancer genome atlas
GEO: Gene expression omnibus
KEGG: Kyoto encyclopedia of genes and genomes

## References

[1] Siegel RL, Miller KD, Jemal A (2019). Cancer statistics, 2019. Cancer J Clin.

[2] Ljungberg B, Campbell SC, Choi HY, Jacqmin D, Lee JE, Weikert S (2011). The epidemiology of renal cell carcinoma. Eur Urol.

[3] Patil S, Manola J, Elson P, Negrier S, Escudier B, Eisen T (2012). Improvement in overall survival of patients with advanced renal cell carcinoma: prognostic factor trend analysis from an international data set of clinical trials. J Urol.

[4] Molina AM, Lin X, Korytowsky B, Matczak E, Lechuga MJ, Wiltshire R (2014). Sunitinib objective response in metastatic renal cell carcinoma: analysis of 1059 patients treated on clinical trials. Eur J Cancer (Oxford, England: 1990).

[5] Li X, Ma S, Yi C (2016). Pseudouridine: the fifth RNA nucleotide with renewed interests. Curr Opin Chem Biol.

[6] Jack K, Bellodi C, Landry DM, Niederer RO, Meskauskas A, Musalgaonkar S (2011). rRNA pseudouridylation defects affect ribosomal ligand binding and translational fidelity from yeast to human cells. Mol Cell.

[7] Martinez NM, Su A, Burns MC, Nussbacher JK, Schaening C, Sathe S (2022). Pseudouridine synthases modify human pre-mRNA co-transcriptionally and affect pre-mRNA processing. Mol Cell.

[8] Kierzek E, Malgowska M, Lisowiec J, Turner DH, Gdaniec Z, Kierzek R (2014). The contribution of pseudouridine to stabilities and structure of RNAs. Nucleic Acids Res.

[9] Carlile TM, Rojas-Duran MF, Zinshteyn B, Shin H, Bartoli KM, Gilbert WV (2014). Pseudouridine profiling reveals regulated mRNA pseudouridylation in yeast and human cells. Nature.

[10] Nombela P, Miguel-López B, Blanco S (2021). The role of m(6)A, m(5)C and Ψ RNA modifications in cancer: novel therapeutic opportunities. Mol Cancer.

[11] Cui Q, Yin K, Zhang X, Ye P, Chen X, Chao J (2021). Targeting PUS7 suppresses tRNA pseudouridylation and glioblastoma tumorigenesis. Nat cancer.

[12] Kan G, Wang Z, Sheng C, Chen G, Yao C, Mao Y (2021). Dual inhibition of DKC1 and MEK1/2 synergistically restrains the growth of colorectal cancer cells. Adv Sci (Weinheim, Baden-Wurttemberg, Germany).

[13] Deogharia M, Mukhopadhyay S, Joardar A, Gupta R (2019). The human ortholog of archaeal Pus10 produces pseudouridine 54 in select tRNAs where its recognition sequence contains a modified residue. RNA (New York, NY).

[14] Song D, Guo M, Xu S, Song X, Bai B, Li Z (2021). HSP90-dependent PUS7 overexpression facilitates the metastasis of colorectal cancer cells by regulating LASP1 abundance. J Exp Clin Cancer Res CR.

[15] Hou P, Shi P, Jiang T, Yin H, Chu S, Shi M (2020). DKC1 enhances angiogenesis by promoting HIF-1α transcription and facilitates metastasis in colorectal cancer. Br J Cancer.

[16] Aza-Blanc P, Cooper CL, Wagner K, Batalov S, Deveraux QL, Cooke MP (2003). Identification of modulators of TRAIL-induced apoptosis via RNAi-based phenotypic screening. Mol Cell.

[17] Song J, Zhuang Y, Zhu C, Meng H, Lu B, Xie B (2020). Differential roles of human PUS10 in miRNA processing and tRNA pseudouridylation. Nat Chem Biol.

[18] Lu TX, Rothenberg ME (2018). MicroRNA. J Allergy Clin Immunol.

[19] Schirle NT, Sheu-Gruttadauria J, MacRae IJ (2014). Structural basis for microRNA targeting. Science (New York NY).

[20] Ambros V (2004). The functions of animal microRNAs. Nature.

[21] Di Leva G, Garofalo M, Croce CM (2014). MicroRNAs in cancer. Annu Rev Pathol.

[22] Rupaimoole R, Slack FJ (2017). MicroRNA therapeutics: towards a new era for the management of cancer and other diseases. Nat Rev Drug Discov.

[23] Hill L, Browne G, Tulchinsky E (2013). ZEB/miR-200 feedback loop: at the crossroads of signal transduction in cancer. Int J Cancer.

[24] Xiao W, Lou N, Ruan H, Bao L, Xiong Z, Yuan C (2017). Mir-144-3p promotes cell proliferation, metastasis, sunitinib resistance in clear cell renal cell carcinoma by downregulating ARID1A. Cell Physiol Biochem.

[25] Lokeshwar SD, Talukder A, Yates TJ, Hennig MJP, Garcia-Roig M, Lahorewala SS (2018). Molecular characterization of renal cell carcinoma: a potential Three-MicroRNA prognostic signature. Cancer Epidemiol Biomark Prev.

[26] Zhang J, Ye Y, Chang DW, Lin SH, Huang M, Tannir NM (2018). Global and targeted miRNA expression profiling in Clear Cell Renal Cell Carcinoma Tissues potentially Links miR-155-5p and mir-210-3p to both tumorigenesis and recurrence. Am J Pathol.

[27] Quail DF, Joyce JA (2013). Microenvironmental regulation of tumor progression and metastasis. Nat Med.

[28] Wicks EE, Semenza GL (2022). Hypoxia-inducible factors: cancer progression and clinical translation. J Clin Investig..

[29] Forsythe JA, Jiang BH, Iyer NV, Agani F, Leung SW, Koos RD (1996). Activation of vascular endothelial growth factor gene transcription by hypoxia-inducible factor 1. Mol Cell Biol.

[30] Schödel J, Grampp S, Maher ER, Moch H, Ratcliffe PJ, Russo P (2016). Hypoxia, hypoxia-inducible transcription factors, and Renal Cancer. Eur Urol.

[31] Guzzi N, Cieśla M, Ngoc PCT, Lang S, Arora S, Dimitriou M (2018). Pseudouridylation of tRNA-derived fragments steers translational control in stem cells. Cell.

[32] von Roemeling CA, Radisky DC, Marlow LA, Cooper SJ, Grebe SK, Anastasiadis PZ (2014). Neuronal pentraxin 2 supports clear cell renal cell carcinoma by activating the AMPA-selective glutamate receptor-4. Cancer Res.

[33] Jana S, Hsieh AC, Gupta R (2017). Reciprocal amplification of caspase-3 activity by nuclear export of a putative human RNA-modifying protein, PUS10 during TRAIL-induced apoptosis. Cell Death Dis.

[34] Wotschofsky Z, Liep J, Meyer HA, Jung M, Wagner I, Disch AC (2012). Identification of metastamirs as metastasis-associated microRNAs in clear cell renal cell carcinomas. Int J Biol Sci.

[35] Seitz H, Zamore PD (2006). Rethinking the microprocessor. Cell.

[36] Zhang C, Zhang W, Lu Y, Yan X, Yan X, Zhu X (2016). NudC regulates actin dynamics and ciliogenesis by stabilizing cofilin 1. Cell Res.

[37] Bravo-Cordero JJ, Magalhaes MA, Eddy RJ, Hodgson L, Condeelis J (2013). Functions of cofilin in cell locomotion and invasion. Nat Rev Mol Cell Biol.

[38] Choueiri TK, Kaelin WG (2020). Targeting the HIF2-VEGF axis in renal cell carcinoma. Nat Med.

[39] Qiu B, Ackerman D, Sanchez DJ, Li B, Ochocki JD, Grazioli A (2015). HIF2α-dependent lipid storage promotes endoplasmic reticulum homeostasis in clear-cell renal cell carcinoma. Cancer Discov.

[40] Baldewijns MM, van Vlodrop IJ, Vermeulen PB, Soetekouw PM, van Engeland M, de Bruïne AP (2010). VHL and HIF signalling in renal cell carcinogenesis. J Pathol.

[41] Karijolich J, Yi C, Yu YT (2015). Transcriptome-wide dynamics of RNA pseudouridylation. Nat Rev Mol Cell Biol.

[42] Guzzi N, Muthukumar S, Cieśla M, Todisco G, Ngoc PCT, Madej M (2022). Pseudouridine-modified tRNA fragments repress aberrant protein synthesis and predict leukaemic progression in myelodysplastic syndrome. Nat Cell Biol.

[43] Gurha P, Gupta R (2008). Archaeal Pus10 proteins can produce both pseudouridine 54 and 55 in tRNA. RNA (New York NY).

[44] Wang Y, Zhang Z, He H, Song J, Cui Y, Chen Y (2023). Aging-induced pseudouridine synthase 10 impairs hematopoietic stem cells. Haematologica.

[45] Ramp U, Caliskan E, Mahotka C, Krieg A, Heikaus S, Gabbert HE (2003). Apoptosis induction in renal cell carcinoma by TRAIL and gamma-radiation is impaired by deficient caspase-9 cleavage. Br J Cancer.

[46] Alarcón CR, Lee H, Goodarzi H, Halberg N, Tavazoie SF (2015). N6-methyladenosine marks primary microRNAs for processing. Nature.

[47] Peng W, Li J, Chen R, Gu Q, Yang P, Qian W (2019). Upregulated METTL3 promotes metastasis of colorectal Cancer via miR-1246/SPRED2/MAPK signaling pathway. J Exp Clin Cancer Res CR.

[48] Han J, Wang JZ, Yang X, Yu H, Zhou R, Lu HC (2019). METTL3 promote tumor proliferation of bladder cancer by accelerating pri-miR221/222 maturation in m6A-dependent manner. Mol Cancer.

[49] Pandolfini L, Barbieri I, Bannister AJ, Hendrick A, Andrews B, Webster N (2019). METTL1 promotes let-7 MicroRNA Processing via m7G methylation. Mol Cell.

[50] Liu Y, Yang C, Zhao Y, Chi Q, Wang Z, Sun B (2019). Overexpressed methyltransferase-like 1 (METTL1) increased chemosensitivity of colon cancer cells to cisplatin by regulating miR-149-3p/S100A4/p53 axis. Aging.

[51] Xiang X, Fischer R (2004). Nuclear migration and positioning in filamentous fungi. Fung Genet Biol.

[52] Aumais JP, Williams SN, Luo W, Nishino M, Caldwell KA, Caldwell GA (2003). Role for NudC, a dynein-associated nuclear movement protein, in mitosis and cytokinesis. J Cell Sci.

[53] Morris SM, Albrecht U, Reiner O, Eichele G, Yu-Lee LY (1998). The lissencephaly gene product Lis1, a protein involved in neuronal migration, interacts with a nuclear movement protein, NudC. Curr Biol CB.

[54] Liu M, Xu Z, Zhang C, Yang C, Feng J, Lu Y (2021). NudC L279P mutation destabilizes Filamin A by inhibiting the Hsp90 chaperoning pathway and suppresses cell migration. Front cell Dev Biol.

[55] Pollard TD, Cooper JA (2009). Actin, a central player in cell shape and movement. Science (New York NY).

[56] Bamburg JR (1999). Proteins of the ADF/cofilin family: essential regulators of actin dynamics. Annu Rev Cell Dev Biol.

[57] Collazo J, Zhu B, Larkin S, Martin SK, Pu H, Horbinski C (2014). Cofilin drives cell-invasive and metastatic responses to TGF-β in prostate cancer. Cancer Res.

[58] Quintela-Fandino M, Arpaia E, Brenner D, Goh T, Yeung FA, Blaser H (2010). HUNK suppresses metastasis of basal type breast cancers by disrupting the interaction between PP2A and cofilin-1. Proc Natl Acad Sci USA.

[59] Turcotte S, Desrosiers RR, Béliveau R (2003). HIF-1alpha mRNA and protein upregulation involves rho GTPase expression during hypoxia in renal cell carcinoma. J Cell Sci.

[60] Xie H, Song J, Godfrey J, Riscal R, Skuli N, Nissim I (2021). Glycogen metabolism is dispensable for tumour progression in clear cell renal cell carcinoma. Nat Metab.

[61] Masoud GN, Li W (2015). HIF-1α pathway: role, regulation and intervention for cancer therapy. Acta Pharm Sin B.

[62] Fallah J, Brave MH, Weinstock C, Mehta GU, Bradford D, Gittleman H (2022). FDA approval summary: Belzutifan for von Hippel-Lindau Disease-Associated Tumors. Clin Cancer Res.

