# Supplementary material for: HIF1A-repressed PUS10 regulates NUDC/Cofilin1 dependent renal cell carcinoma migration by promoting the maturation of miR-194-5p
Source: Cell Biosci. 2023 Aug 18;13:153. doi: 10.1186/s13578-023-01094-4 (PMC10439626; doi:10.1186/s13578-023-01094-4)
Supplement: Supplementary file 3 — Additional file 3: Table S3. Primers and antibodies used in this study. [file 13578_2023_1094_MOESM3_ESM.docx]

Primers used for quantitative Real Time-PCR in this study.

| Gene Symbol | Forward primer (5’→3’) | Reverse primer (5’→3’) | |
| --- | --- | --- | --- |
| *PUS10* | CTCACCAAAGGCTGTATGCGCT | CCAAGGAGTTTGTGGTAGATTCC | |
| *GAPDH* | GGAGTCAACGGATTTGGT | GTGATGGGATTTCCATTGAT | |
| *Pri-miR-194* | CAGTGGGGCTGCTGTTATCT | GTAGTCCTGGCTGGTCTGCT | |
| *NudC* | AGACCTGCCCAATTACCGC | GCTCCCCATCAATGATCGCT | |
| *PDHB* | GATGAGAAGGTATTTCTGCT | GAGAAATTGAAGGTCATAAA | |
| *TSPAN7* | CTCATCGGAACTGGCACCACTA | CCTGAAATGCCAGCTACGAGCT | |
| *ITPKB* | GTTCCCCTCAGTGGTACTGC | TGACCCCGTAATTGGCTCAG | |
| *HNRNPA0* | GCTTTGGCTTCGTGACCTAC | AACTGCGAGAAGTGCTCGAT | |
| *PUS10-promoter-HRE1* | GTTCACGCCATTCTCCTG | AGACCATCCTGGCTAACTC | |
| *PUS10-promoter-HRE2* | GTTGTTGTTTGAGATGGAGTC | CAGGAGAATGGCGTGAAC | |
| *PUS10-promoter-HRE3* | ACACAAAGTCCATCCATAGG | ACGCAACAGGAACTCAAG | |
| *PUS10-promoter-HRE4* | CTGATTAGCTGGGACTACA | ATCACGAGGTCAGGAGAT | |
| *PUS10-promoter-HRE5* | GTAGGTGATTTGACATTTGA | TGGTAGTGCTGGACATC | |
| *PUS10-promoter-HRE6* | GCTGCGACAATTAGTTACC | TCTACCTAGATCAGGACAGTT | |
| Primers used for stem-loop RT-PCR of piRNAs | | | |
| Gene Symbol | Stem-loop Reverse transcription primers (5’→3’) | Forward primer (5’→3’) | Reverse primer (5’→3’) |
| *miR-194-5p* | GTCGTATCCAGTGCAGGGTCCGAGGTATTCGCACTGGATACGACTCCACA | CGCGTGTAACAGCAACTCCA | AGTGCAGGGTCCGAGGTATT |
| *U6* | AACGCTTCACGAATTTGCGT | CTCGCTTCGGCAGCACA | AACGCTTCACGAATTTGCGT |

Antibody used in this study

| Antibody | company |
| --- | --- |
| *GAPDH* | *abcam* |
| *PUS10* | *Novus* |
| *DGCR8* | *abcam* |
| *NudC* | *abclonal* |
| *Cofilin1* | *Proteintech* |
| *HIF-1A* | *CST* |
| *HIF-2A* | *CST* |
