# Supplementary material for: HIF1A-repressed PUS10 regulates NUDC/Cofilin1 dependent renal cell carcinoma migration by promoting the maturation of miR-194-5p
Source: Cell Biosci. 2023 Aug 18;13:153. doi: 10.1186/s13578-023-01094-4 (PMC10439626; doi:10.1186/s13578-023-01094-4)
Supplement: Supplementary file 4 — Additional file 4: Figure S1. Downregulation of PUS10 in RCC tissue at protein level in public IHC results. Figure S2. Knock down of PUS10 promotes cancer cell migration but doesn’t influence its proliferation and apoptosis. Figure S3. PUS10 inhibits RCC migration, which is not achieved by its pseudouridine synthase activity. Figure S4. miR-194-5p mediates the impact of PUS10 on RCC migration. Figure S5. NudC was identified as the downstream target of miR-194-5p. Figure S6. NudC/Cofilin1 was involved in PUS10 inducing inhibition on RCC migration. [file 13578_2023_1094_MOESM4_ESM.docx]

­­­
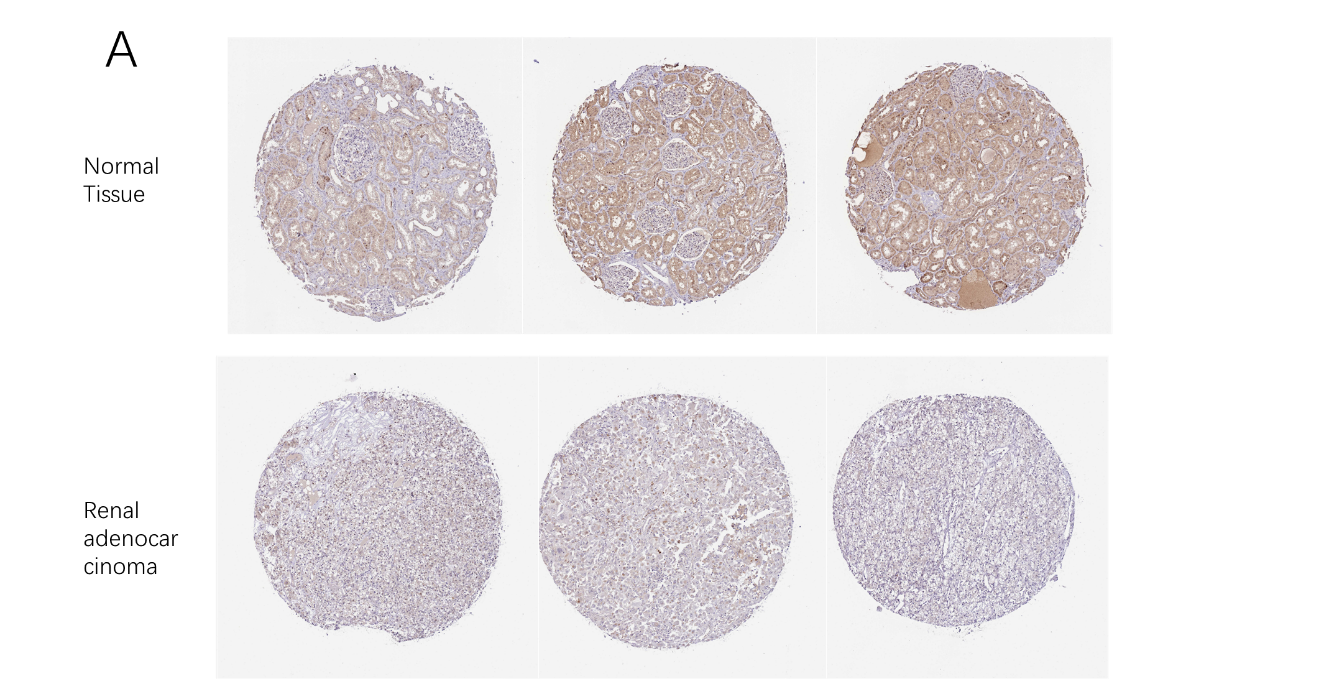


**Figure S1** **Downregulation of PUS10 in RCC tissue at protein level in public IHC results.** **a,** Representative PUS10 staining IHC figures of normal tissue and renal adenocarcinoma from THPA online database.


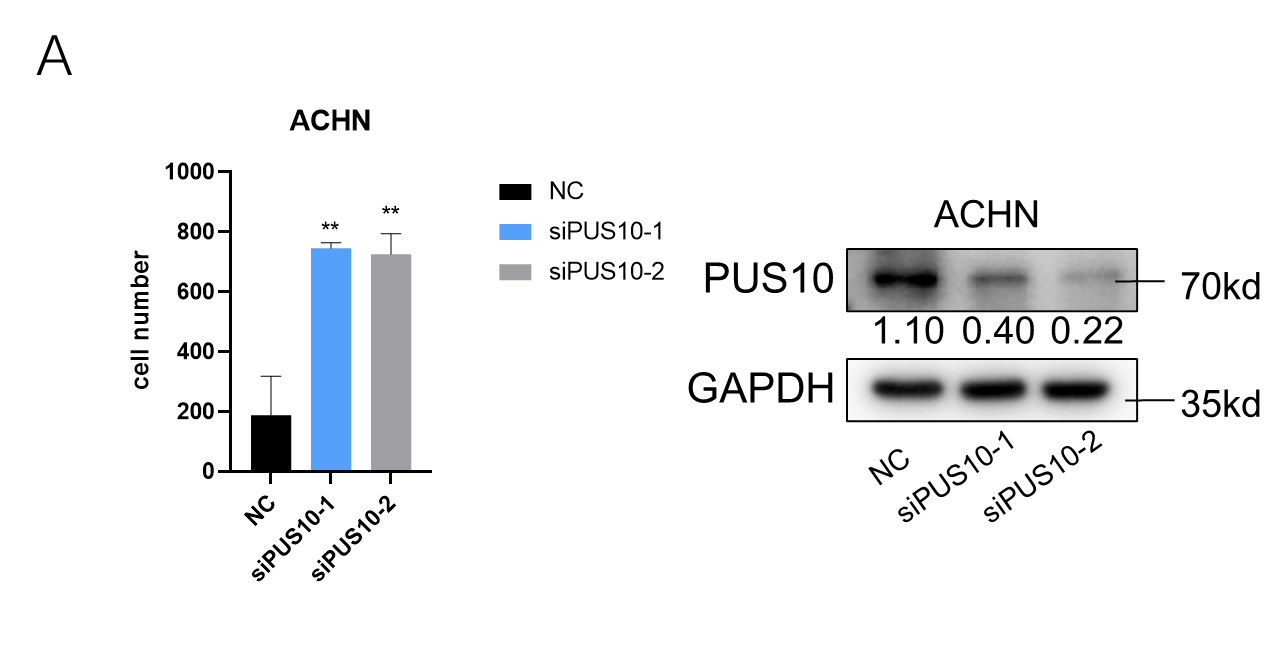


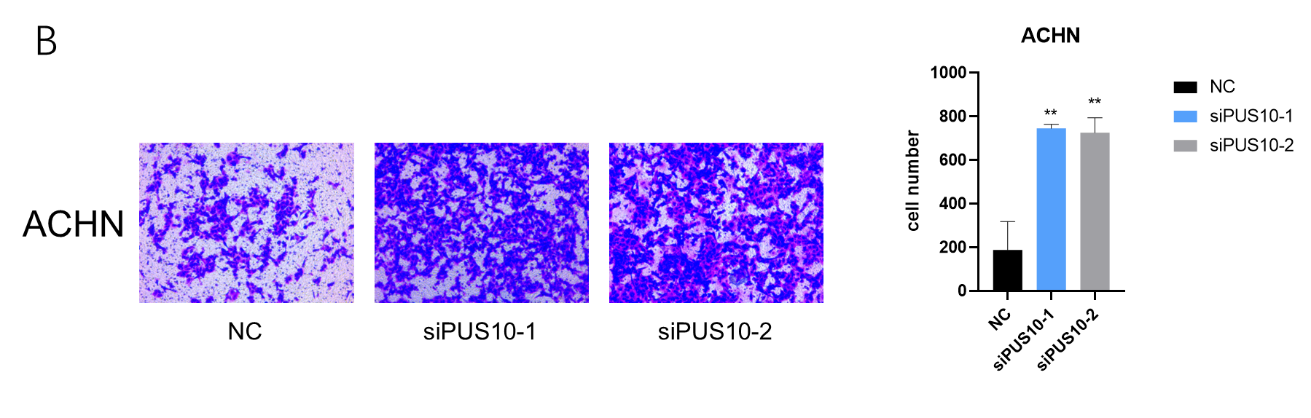


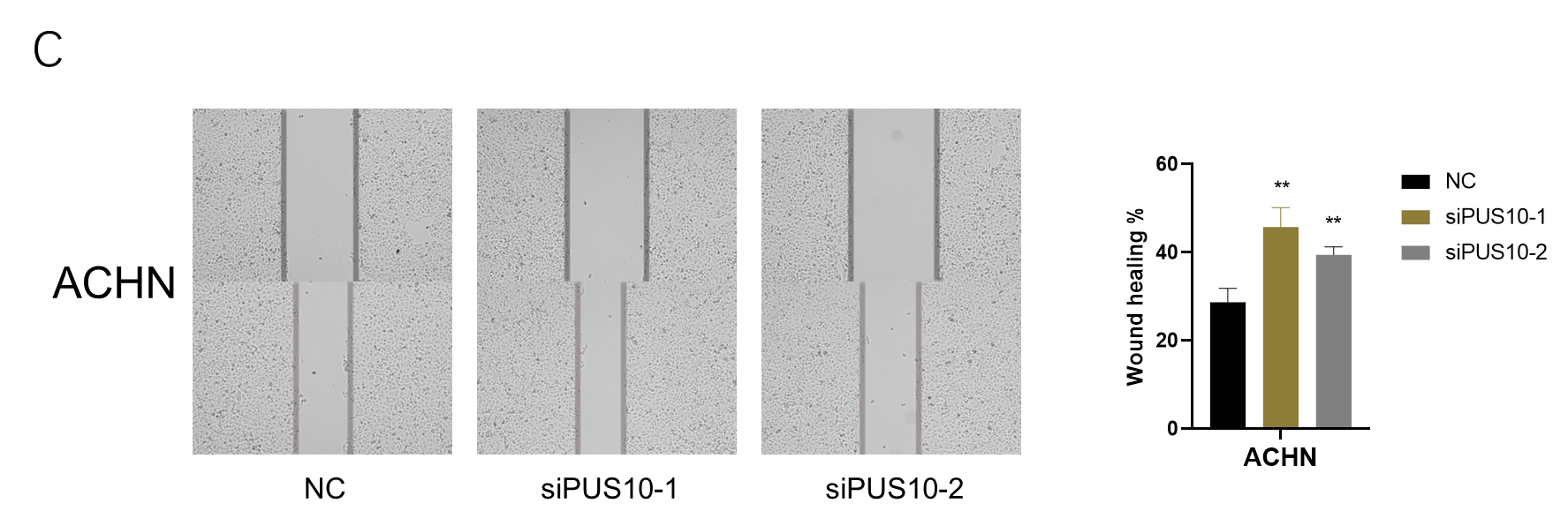


**
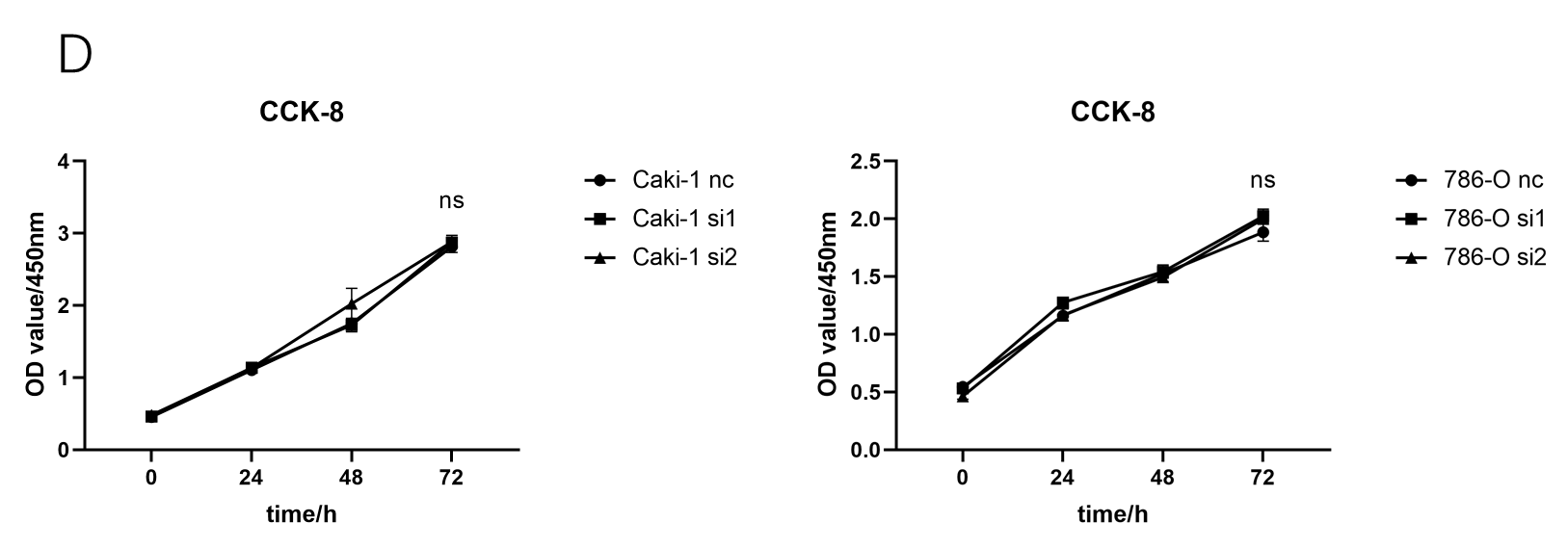
**

**
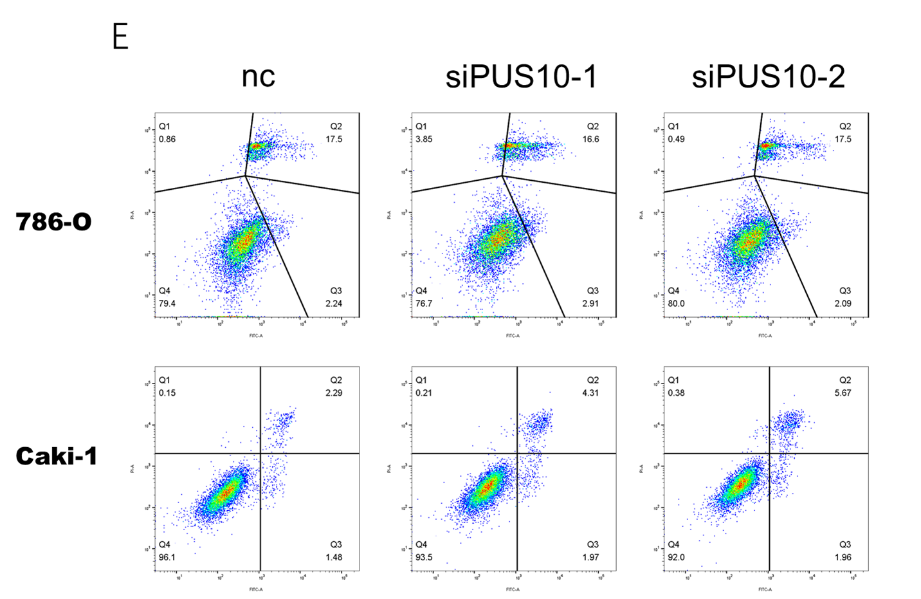
**

**Figure S2 Knock down of PUS10 promotes cancer cell migration but doesn’t influence its proliferation and apoptosis.** a, qRT‒PCR assay and Western blot were performed to estimate the knock down efficiency after the transfection of siRNAs. Three independent experiments are shown as the mean±SD, and GAPDH was used as a reference. b,c, Transwell assays and wound healing assays were performed to assess the migration ability of ACHN after PUS10 silencing. Migrating cells in three replicate experiments were counted and are presented as the mean± SD in the histogram. ­­d, CCK-8 assays shown silencing of PUS10 doesn’t influence the proliferation of 786-O and Caki-1. e, Flow cytometry exhibited depletion of PUS10 have little influence on the apoptosis of RCC cell lines.


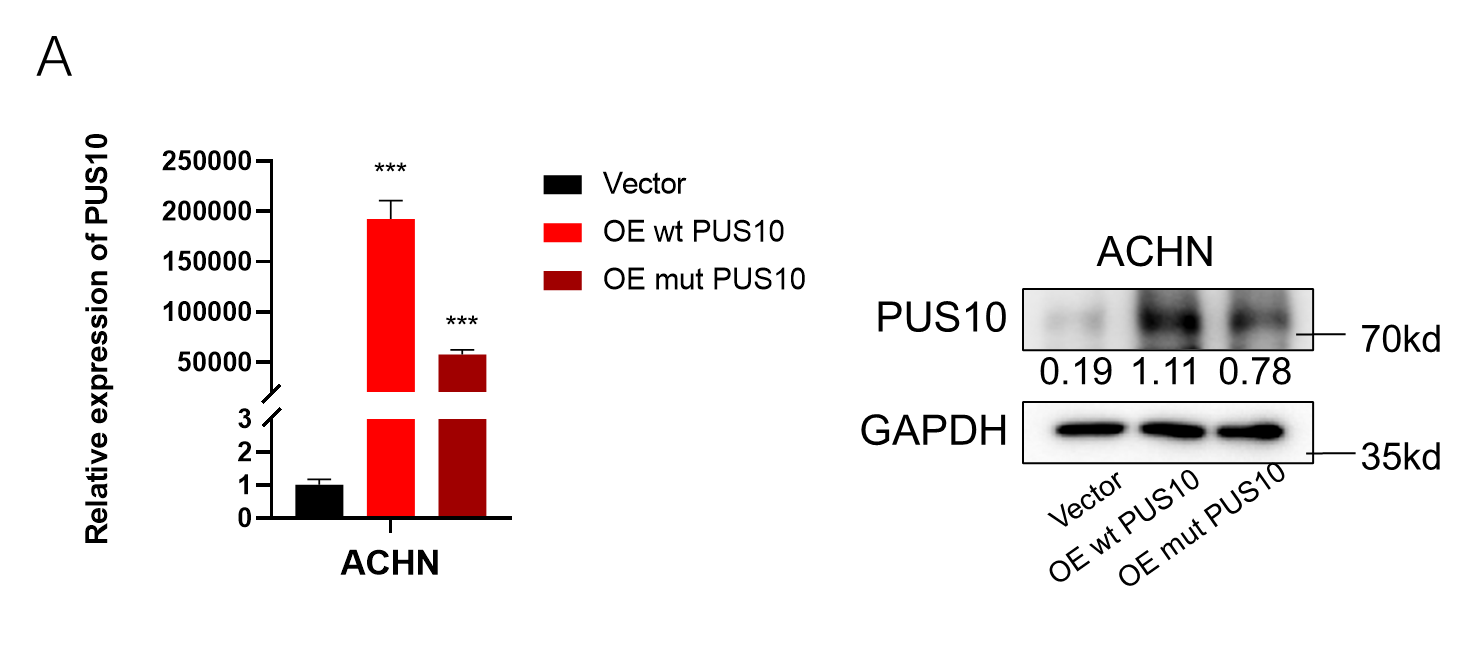

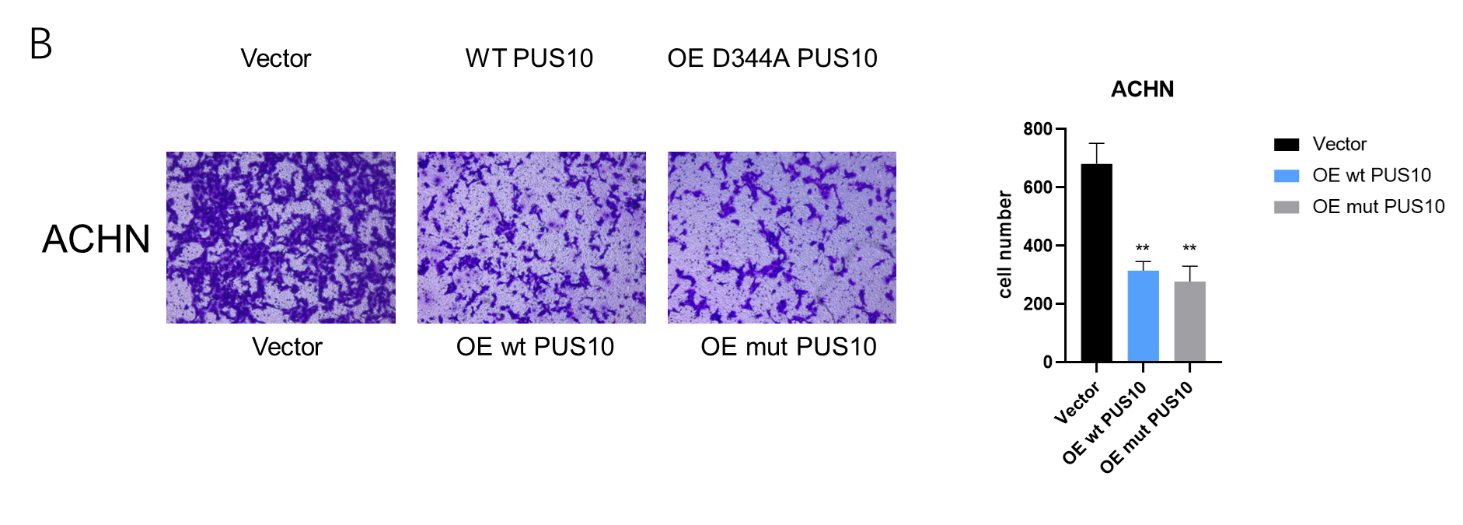


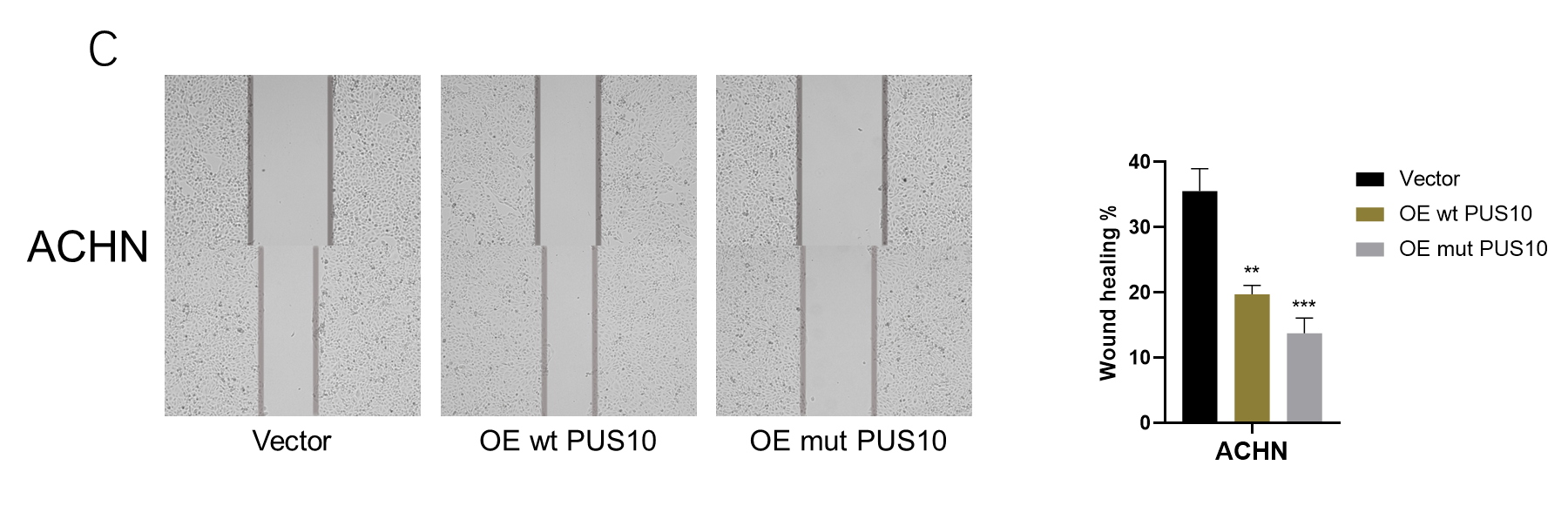


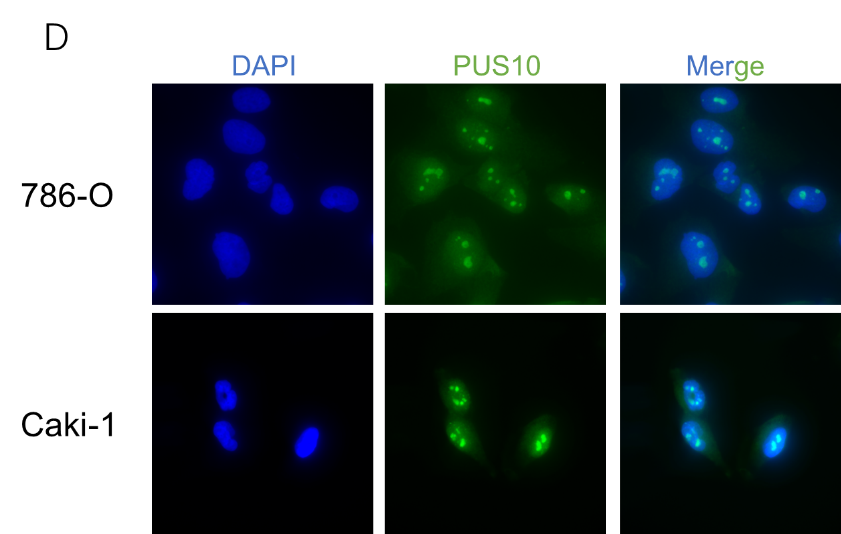


**Figure S3 PUS10 inhibits RCC migration, which is not achieved by its pseudouridine synthase activity.** a, qRT‒PCR assay and Western blot were conducted to confirm the over expression of wt PUS10 and D344A mutant PUS10 in ACHN. Three independent experiments are shown as the mean±SD, and GAPDH was used as a reference. b,c, Transwell assays and wound healing assays indicated the migration of ACHN was suppressed after over expression of both wildtype and mutant PUS10. Migrating cells in three replicate experiments were counted and are presented as the mean± SD in the histogram. d, Immunofluorescence assay were performed to determine the cellular distribution of PUS10 in RCC cells. Endogenous PUS10 protein was in green and nuclei was stained with DAPI (blue).


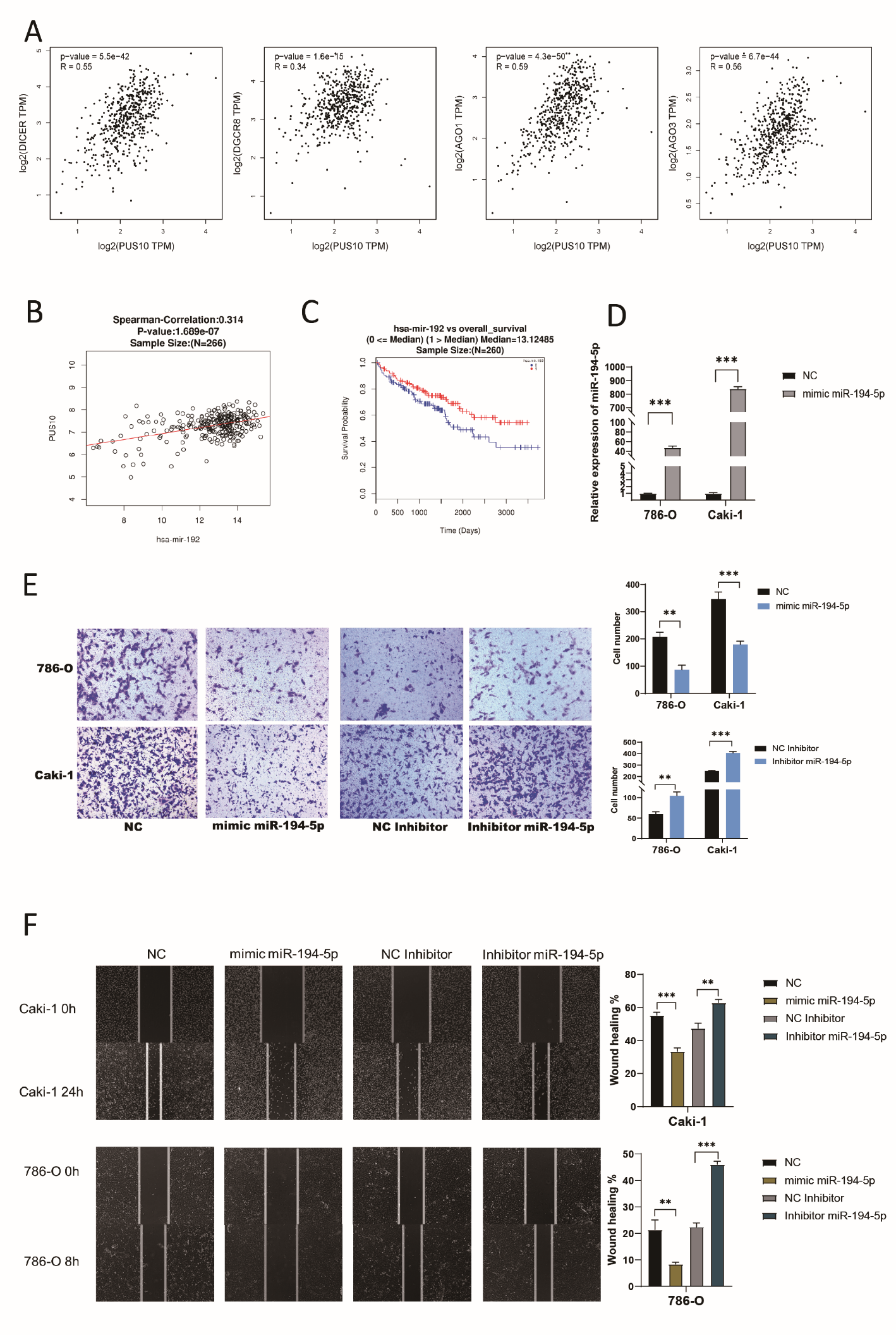
­­­

**Figure. S4 miR-194-5p mediates the impact of PUS10 on RCC migration.** ­­a, positive correlation between the expression of PUS10 and that of microRNA biogenesis associated proteins in KIRC based on TCGA data. b, Positive correlation between the expression of PUS10 and miR-192 in 266 KIRC and its paired normal tissues based on TCGA data. c, Survival analysis based on TCGA data revealed high expression of miR-192 predict good prognosis. ((*r* = 0.31, *P* value < 0.001)). d, qRT-PCR was performed to validate the ectopic expressing of miR-194-5p in RCC. e, Transwell assays demonstrated miR-194-5p inhibit the migration of RCC, migrating cells in three replicated experiments were counted and presented as mean± SD in the histogram. f. Wound healing assay was conducted to verify miR-194-5p inhibit the migration of RCC. A representative of three replicated experimented was exhibited. The wound healing rate is calculated and presented in mean ± S.D. ∗p < 0.05, ∗∗p < 0.01, ∗∗∗p < 0.001; ns, not significant.


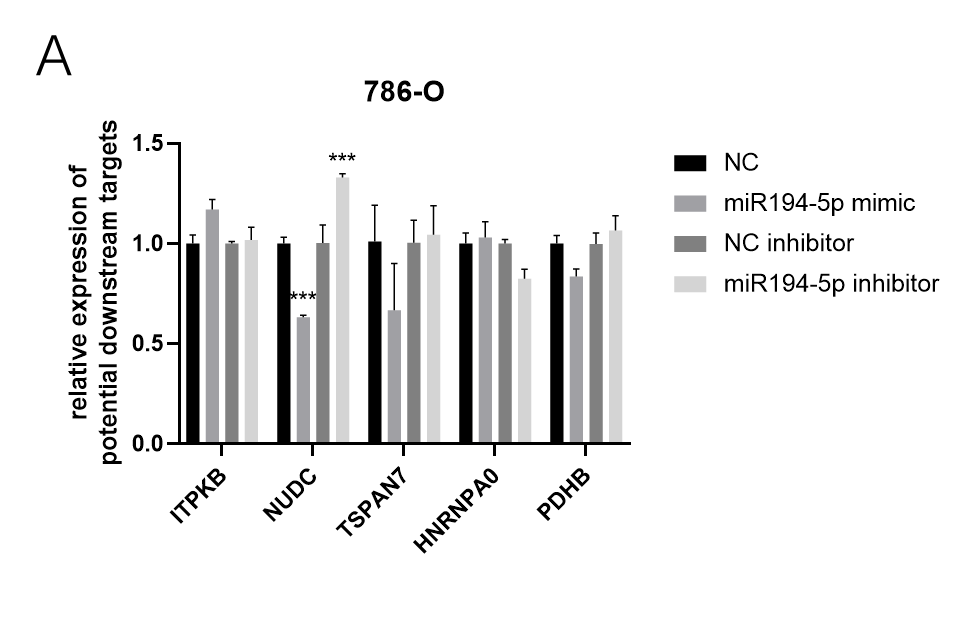


**Figure S5 NudC was identified as the downstream target of miR-194-5p.** a, Changed expression of NudC at mRNA level upon transfection of miR-194-5p mimics and inhibitor in RCC cells are determined in qRT-PCR. Data from three independent experiment is presented in mean± SD in the histogram. ∗p < 0.05, ∗∗p < 0.01, ∗∗∗p < 0.001; ns, not significant.


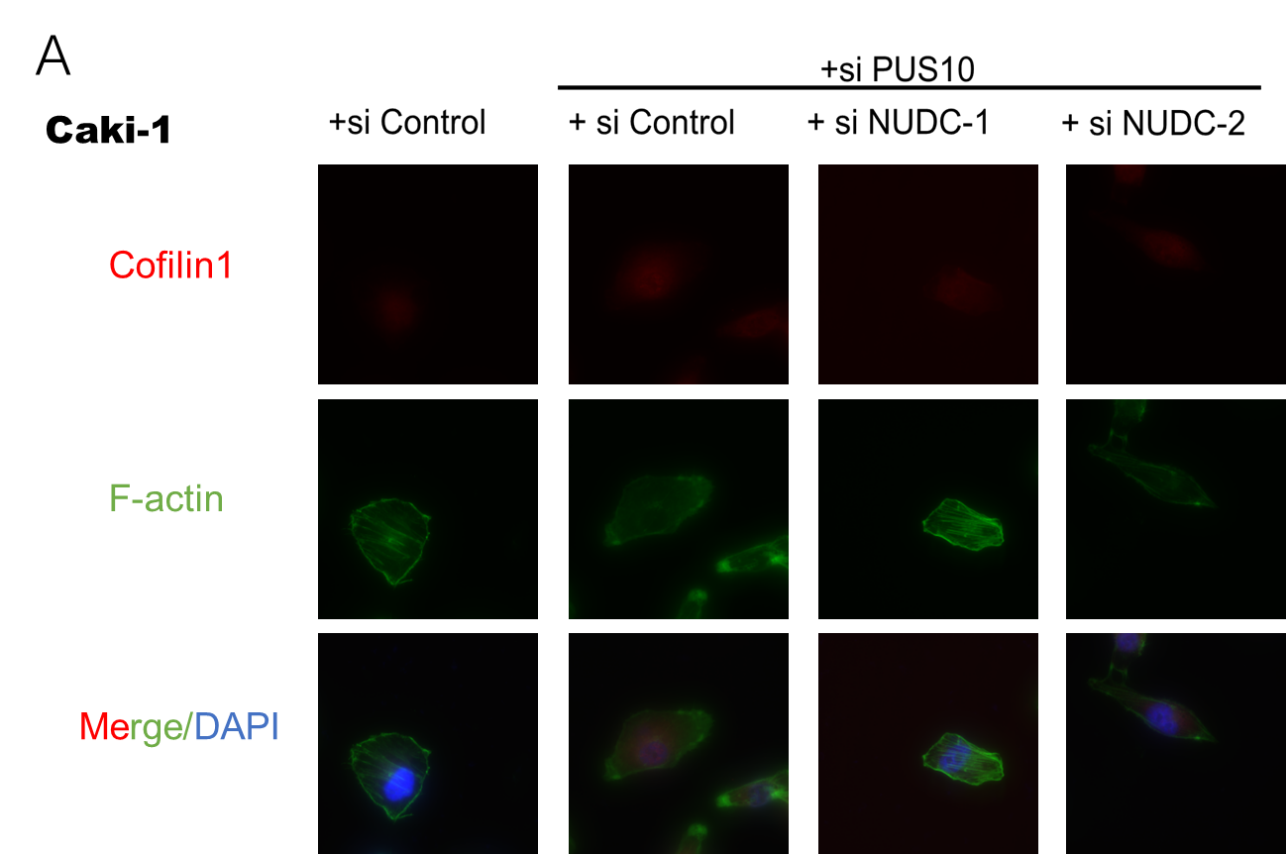
_­­_

**Figure S6 NudC/Cofilin1 was involved in PUS10 inducing inhibition on RCC migration.** a, Representative images of immunostaining using anti-Cofilin1 antibody and Phalloidin are exhibited in Caki-1 cells, showing the impact of PUS10 on Cofilin1 ­dependent cytoskeleton.­
